# Treatment outcomes of pre-surgical infant orthopedics in patients with non-syndromic cleft lip and/or palate: A systematic review and meta-analysis of randomized controlled trials

**DOI:** 10.1371/journal.pone.0181768

**Published:** 2017-07-24

**Authors:** Hamid Reza Hosseini, Eleftherios G. Kaklamanos, Athanasios E. Athanasiou

**Affiliations:** Hamdan Bin Mohammed College of Dental Medicine, Mohammed Bin Rashid University of Medicine and Health Sciences, Dubai, United Arab Emirates; College of Dentistry, The University of Iowa, UNITED STATES

## Abstract

**Background:**

Non-syndromic clefts lip and/or palate (CL/P) defects may have manifold significant and detrimental consequences for the affected individuals and their family environment. Although the use of pre-surgical infant orthopedics (PSIO) was introduced as a means to improve management and treatment outcomes, there still remains a controversy.

**Objective:**

To investigate the effectiveness of PSIO in patients with non-syndromic CL/P and evaluate the quality of the available evidence.

**Search methods:**

Search without restrictions, together with hand searching, until May 2016.

**Selection criteria:**

Randomized clinical trials investigating the effects of pre-surgical infant orthopedic appliances.

**Data collection and analysis:**

Following study retrieval and selection, data extraction and individual study risk of bias assessment using the Cochrane Risk of Bias Tool took place. The overall quality of the available evidence was assessed with the Grades of Recommendation, Assessment, Development and Evaluation approach.

**Results:**

Finally 20 papers (3 unique trials) were identified, involving a total of 118 patients with unilateral complete CL/P and 16 with cleft of the soft and at least two thirds of the hard palate. Eight publications were considered as being of low, four of unclear and eight of high risk of bias. In general, the investigated appliances did not present significant effects when compared to each other or to no treatment in terms of feeding and general body growth, facial esthetics, cephalometric variables, maxillary dentoalveolar variables and dental arch relationships, speech and language evaluation, caregiver-reported outcomes, economic evaluation, as well as, adverse effects and problems. Overall, the quality of the available evidence was considered low.

**Conclusions:**

The aforementioned findings could provide initial guidance in the clinical setting. However, given the multitude of parameters, which may have affected the results, good practice would suggest further research, in order to reach more robust relevant recommendations for management decisions in individual cases.

## Introduction

### Rationale

Clefts of the upper lip, alveolar ridge and palate are considered to be one of the most common congenital malformations in humans [1]. These defects, involving various soft and osseous tissues of the oral cavity, occur when the morphogenesis of the upper lip and the palate taking place between the 6^th^ and the 12^th^ week of fetal development, deviates from normal and the fusion between the maxillary with the two medial nasal prominences followed by the primary palate with the two lateral palatal shelves is disrupted [2]. Such cleft defects may have manifold significant and detrimental consequences for the affected individual and their family environment when considering its potential impact on normal feeding and bodily growth, proper hearing and speech development, harmonious growth of the face and occlusion together with the various psychosocial parameters associated with the deformative nature of the condition [3].

The use of pre-surgical infant orthopedics (PSIO) was introduced as a means to improve management and treatment outcomes of patients with cleft lip and/or palate in the short-term by assisting feeding, preventing delays in development and helping in normalizing the function of deglutition. In addition, the resulting amelioration in the distorted dental arch and nasal forms also facilitates surgical procedures by minimizing tension at the surgical site [4]. In the long-term, it has been suggested that speech development, maxillofacial growth and facial esthetics are enhanced, thus decreasing the need for specialist intervention in the future [5].

During the last forty years, PSIO procedures became integrated into the comprehensive care protocols for patients with clefts in many teams around the world [5–8]. Interestingly however, this practice still remains a subject of controversy [9]. Two systematic reviews evaluating PSIO in general [10, 11], and one assessing growth and development outcomes after various feeding interventions in infants with cleft lip and/or palate [12] have been published recently. However, neither of these attempted to summarize the available quality of evidence and thus provide an insight into the strength of the relevant recommendations.

### Objectives

The aim of the present paper was to systematically investigate the effectiveness of pre-surgical infant orthopedics in patients with non-syndromic cleft lip and/or palate and evaluate the quality of the available evidence.

The following null hypotheses were tested:

1. There is no difference between the outcomes of PSIO compared no treatment.
2. There is no difference in the comparative effectiveness of various PSIO appliances and modalities.

## Materials and methods

### Protocol and registration

The present review was based on a specific protocol developed and piloted following the guidelines outlined in the PRISMA-P statement [13] and registered in PROSPERO (CRD42016047940) (S1 File). Furthermore conduct and reporting followed the Cochrane Handbook for Systematic Reviews of Interventions [14] and the PRISMA statement [15], respectively.

### Eligibility criteria

The eligibility criteria, based on the PICOS (Participants, Intervention, Comparison, Outcomes, Study design) acronym, were as follows:

*Participants*: children of any age with any kind of non-syndromic cleft lip and/or palate defect.

*Intervention*: any type of PSIO appliance.

*Comparison*: no treatment or an alternative PSIO protocols.

*Outcomes*: Any outcome relevant to PSIO appliance treatment.

*Study design*: Randomized Clinical Trials (RCTs). Animal studies, non-comparative studies (case reports and case series), systematic reviews and meta-analyses were excluded.

### Information sources and search strategy

One author (HRH) developed detailed search strategies for each database searched. These were based on the strategy developed for MEDLINE but revised appropriately for each database to take account of the differences in controlled vocabulary and syntax rules (S1 Table).

No restriction was placed on the language, date or status of publication. In addition, efforts were made to obtain conference proceedings and abstracts where possible and the reference lists of all eligible studies for additional records were searched. The authors of possibly identified ongoing studies were to be contacted in order to provide additional data for the review if available.

### Study selection

Two authors (HRH and EGK) assessed the retrieved records for inclusion independently. They were not blinded to the identity of the authors, their institution, or the results of the research. They obtained and assessed, again independently, the full report of records considered by either reviewer to meet the inclusion criteria. Disagreements were resolved by discussion or consultation with the third author (AEA). A record of all decisions on study identification was kept.

### Data collection and data items

The same two persons performed data extraction independently and any disagreements were again resolved by discussion or consultation with the third author. Data collection forms were used to record the desired information, including bibliographic details of the study, details on study design and verification of study eligibility, participant characteristics (where available number, age, gender), intervention characteristics (PSIO appliance used, PSIO treatment protocol), details on outcomes assessed and assessment procedures and any additional information of interest (a prior sample size calculation, methodology reliability assessment and data on compliance with the assigned intervention protocol).

The outcomes relevant to PSIO appliance treatment retrieved from the studies included in the present review were categorized as follows: feeding characteristics and nutritional status, facial esthetics, dentofacial cephalometric variables, maxillary dentoalveolar variables, dental arch relationships, hearing, speech and language evaluation, patient and caregiver-reported outcomes, economic evaluation related outcomes, and finally, adverse effects and problems related to PSIO appliances and procedures.

### Risk of bias in individual studies

Two authors (HRH and EGK) assessed the risk of bias in the included studies, independently and in duplicate during the data extraction process, using The Cochrane Collaboration’s Risk of Bias assessment tool for RCTs [15]. Any disagreements were to be resolved by discussion or consultation with the third author (AEA).

### Summary measures and synthesis of results

In situations where the retrieved data used different indices measuring the same concept on different scales with a high degree of correlation, the effects of the interventions were planned to be expressed as standardized values (i.e. the Standardized Mean Difference (SMD) together with the relevant 95% Confidence Interval (CI)), in order to enable quantitative synthesis [16]. In case that in a particular comparison only one index was recorded, the intervention effect was planned to be expressed as the Weighted Mean Difference (WMD) together with the 95% CI.

The random effects method for meta-analysis was to be used to combine data from studies that reported similar measurements in appropriate statistical forms [17, 18], since they were expected to differ across studies due to clinical diversity, in terms of participant and intervention characteristics.

To identify the presence and extent of between-study heterogeneity, the overlap of the 95% CI for the results of individual studies was to be inspected graphically, and the I^2^ statistic were to be calculated [15].

All analyses were to be carried out with Comprehensive Meta-analysis software 2.2.046 (Biostat Inc.). Significance (a) was set at 0.05, except for 0.10 used for the heterogeneity tests [19].

### Risk of bias across studies and additional analyses

If deemed possible, exploratory subgroup analyses were planned according to participant and intervention characteristics. In addition, if a sufficient number of trials were identified, analyses were planned for “small-study effects” and publication bias [15].

In addition, the quality of evidence and strength of recommendations at longest follow up available for key outcomes of the systematic review were ultimately to be assessed based on the Grades of Recommendation, Assessment, Development and Evaluation (GRADE) approach [20].

## Results

### Study selection

**Fig 1:**
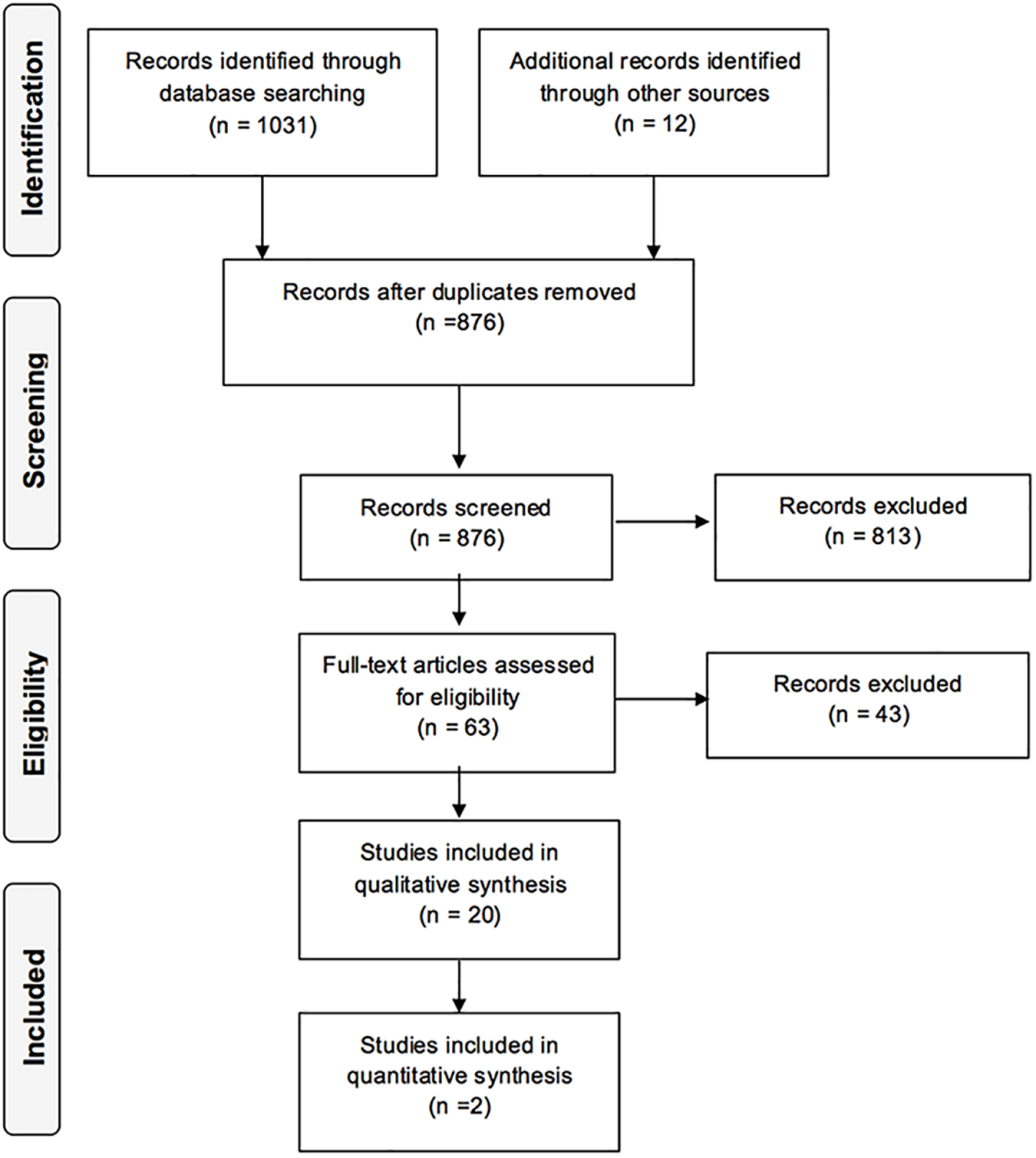
Flowchart of records through the reviewing process.

The flowchart of records through the reviewing process is shown in Fig 1. Initially 1043 records were identified, and 167 were excluded as duplicates and 813 more on the basis of their title and abstract. Finally, 20 full-text reports were included in the systematic review [5, 8, 21–38]. Most data in the Konst thesis [24] were included in subsequent publications Konst et al., [22, 23, 25–30] and in the context of the present review it was considered only for the outcome “future need of speech therapy”.

### Study characteristics

The characteristics of the studies included in the present systematic review are presented in S2–S5 Tables. The papers, which were published between 1998 and 2015, recruited 118 patients with unilateral complete cleft lip and palate (UCLP) and 16 with cleft of the soft and at least two thirds of the hard palate. No studies with children with bilateral cleft lip and palate were retrieved.

Eighteen of the eligible publications comprised a part of a larger trial, the DUTCHCLEFT, that was conducted in three academic cleft palate centers in the Netherlands, enrolled in total 54 patients (41 boys and 13 girls) with non-syndromic UCLP from 1993 to 1996 and compared the effect of a passive PSIO appliance without external retention to no treatment [5, 21–38]. Subjects randomized into the intervention group, received treatment commencing within 2 weeks post partum till surgical closure of the soft palate (around 52 weeks). The appliance was worn 24 h per day, except for cleaning, and was adjusted, repaired or replaced as needed. Check-up appointments were scheduled every 3 weeks until surgical lip closure (around 18 weeks) and every 4 to 6 weeks thereafter until surgical closure of the soft palate. Subjects belonging to the no-treatment control group did not wear any kind of appliance and were scheduled for appointments at age 6 weeks after birth, as well as, before and after surgical closure of the lip and the soft palate defects.

Two of the 27 children of the intervention group hardly used the appliances, and in one case the appliance was worn for 78 weeks by mistake. These infants remained in their assigned group according to the intention to treat rationale. No other data on whether the plates were worn by every patient as indicated were given.

In the context of the DUTCHCLEFT, in various subsamples from the initial patient group and at various time points until the age of 12 years, the following domains were evaluated: feeding characteristics and nutritional status [31]; facial esthetics [33, 34]; dentofacial cephalometric variables [36]; maxillary dentoalveolar variables [5, 32]; dental arch relationships [29, 38]; hearing, speech and language evaluation [22–27]; patient and caregiver-reported outcomes [35]; economic evaluation related outcomes [5, 21, 30].

The Masarei et al. study [8], which was conducted in the UK and followed a slightly different surgical protocol, involved 33 infants (21 boys and 12 girls) with non-syndromic UCLP and 16 infants (9 boys and 7 girls) with cleft of the soft palate accompanied by at least two thirds of the hard palate. The study compared an active PSIO appliance (with a stainless steel spring permitting active approximation of the cleft segments) without external retention to no treatment in the former group and a passive PSIO appliance without external retention to no treatment in the latter. Subjects randomized into the intervention group, received treatment commencing within 2 weeks post partum till surgical closure of the soft palate (around 6 months). The appliance was worn 24 h per day, except for cleaning, and was adjusted or replaced as needed. Unilateral complete cleft lip and palate subjects were scheduled for check-up appointments 5 or 6 times within the first 3 months (surgical closure of the lip and the anterior palate performed as closely as possible to 12 weeks of age) and another 3 before surgical closure of the soft palate. Patients with cleft palate were seen four times during the first 6 months. Initially, most of infants (23 out of 25) in the intervention group complied fully with the indicated protocol of appliance wear. However, compliance decreased over time, and only 14 infants were fully compliant during the intervention period and another subject wore the appliance for 12 hours a day.

The following domains were evaluated, at 3 months of age (prior to any surgical intervention) and 12 months of age (approximately 6 months after soft palate surgical repair): feeding characteristics and nutritional status, economic evaluation related outcomes and adverse effects and problems related to PSIO appliances and procedures.

Finally, the Chang et al. study [37] from Taiwan enrolled 30 infants (19 boys and 11 girls) with complete unilateral cleft lip and palate from May 2010 to March 2013 and compared the effect of the modified Figueroa to the modified Grayson nasolaveolar molding technique. The Grayson technique uses a nasoalveolar molding plate with an anterior extension from which a combination of tapes is applied to help in stabilization. In the Figueroa technique there is no anterior extension and the appliance is held in place only with denture adhesive.

Subjects received treatment commencing within 2 weeks after birth till surgical closure of the lip (around 3 months). The appliance was worn 24 h per day, except for cleaning, and was adjusted every 1 to 2 weeks as needed. All patients completed the trial and compliance was characterized as being exceptionally high. The following domains were evaluated during the initial visit, after nasoalveolar molding but before lip closure surgery, 1 week after surgery and 6 months after surgery: facial esthetics, economic evaluation related outcomes and adverse effects and problems related to PSIO appliances and procedures.

### Risk of bias within studies

Eight studies were considered as being of low risk of bias [5, 28, 29, 32–34, 36, 37], four of unclear risk [8, 31, 35, 38] and eight of high risk [21–27, 30].

In general, all studies included in the present review were considered to present low risk of bias regarding the domains of random sequence generation and allocation concealment. Blinding of the participants, caregivers and the personnel providing the instructions was not feasible. However, in the context of the present research design, there was no reason to suggest that bias could be introduced because of absence of blinding in these cases. Moreover, the review authors did not think that bias could be introduced by the methods described in the publications included in the present review regarding blinding of outcome assessment. The examination of the rest of the domains considered produced varying results that contributed to the variable overall assessment of the included studies.

Table 1 presents the summary findings of the risk of bias assessment for the included studies. More details can be found in S6 and S7 Tables.

**Table 1:** Summary of risk of bias assessment (according to The Cochrane Collaboration’s Risk of Bias assessment tool for RCTs [15]).

	Domain							
Study	1	2	3	4	5	6	7	Summary
**Bongaarts et al., 2004 [29]**	Low	Low	Low	Low	Unclear	Low	Unclear	**Low**
**Bongaarts et al., 2006 [32]**	Low	Low	Low	Low	Unclear	Low	Unclear	**Low**
**Bongaarts et al., 2008 [34]**	Low	Low	Low	Low	Unclear	Low	Unclear	**Low**
**Bongaarts et al., 2009 [36]**	Low	Low	Low	Low	Unclear	Low	Unclear	**Low**
**Chang et al., 2014 [37]**	Low	Low	Low	Low	Low	Low	Low	**Low**
**Konst et al., 1999 [22]**	Low	Low	Low	Low	High	Low	Unclear	**High**
**Konst et al., 2000 [23]**	Low	Low	Low	Low	High	Low	Unclear	**High**
**Konst et al., 2002 [24]**	Low	Low	Low	Low	High	Low	Unclear	**High**
**Konst et al., 2003a [25]**	Low	Low	Low	Low	High	Unclear	Unclear	**High**
**Konst et al., 2003b [26]**	Low	Low	Low	Low	High	Low	Unclear	**High**
**Konst et al., 2003c [27]**	Low	Low	Low	Low	High	Low	Unclear	**High**
**Konst et al., 2004 [30]**	Low	Low	Low	Low	High	Low	Unclear	**High**
**Masarei et al., 2007 [8]**	Low	Low	Low	Low	Unclear	Low	Unclear	**Unclear**
**Noverraz et al., 2015 [38]**	Low	Low	Low	Low	Unclear	Unclear	Unclear	**Unclear**
**Prahl et al., 2001 [5]**	Low	Low	Low	Low	Low	Low	Unclear	**Low**
**Prahl et al., 2003 [28]**	Low	Low	Low	Low	Low	Low	Unclear	**Low**
**Prahl et al., 2005 [31]**	Low	Low	Low	Low	Unclear	Low	Unclear	**Unclear**
**Prahl et al., 2006 [33]**	Low	Low	Low	Low	Low	Low	Unclear	**Low**
**Prahl et al., 2008 [35]**	Low	Low	Low	Low	Unclear	Low	Unclear	**Unclear**
**Severens et al., 1998 [21]**	Low	Low	Low	Low	High	Low	Low	**High**

1: Random sequence generation 2: Allocation concealment, 3: Blinding of participants and personnel, 4: Blinding of outcome assessment, 5: Incomplete outcome data, 6: Selective outcome reporting, 7: Other potential threats to validity

### Effect of pre-surgical infant orthopedics vs. no treatment

The results of the studies included in the present review are presented below. Despite the limited available data, exploratory quantitative syntheses were conducted where possible. Because it was not possible to retrieve a sufficient number of trials, analyses for “small-study effects” and publication bias [15] could not be performed.

#### Feeding characteristics and nutritional status

The effect of pre-surgical infant orthopedics on feeding and nutritional status of cleft lip and/or palate subjects was investigated in two studies eligible for inclusion in the present review [8, 31].

Prahl et al. [31] from the DUTCHCLEFT sample evaluated the ***feeding*** records of in total 47 infants as soon as possible after birth and at 3, 6, 15, 24 weeks of age and observed no significant differences between the two groups regarding time per feeding (min), amount per feeding (mL) and feeding velocity (mL/min). The application of the PSIO appliance, cleft width and initial cleft width explained less than 4.0% of the variance observed in feeding characteristics outcomes. The Masarei et al. [8] study investigated the effect of PSIO appliances in a total of 33 UCLP infants and 16 cleft palate infants and found that no participant from any group was rated as having a normal feeding pattern with the Neonatal Oral Motor Assessment Scale (NOMAS) prior to surgical closure of the soft palate, at three months of age. Similarly, no statistically significant differences were noted regarding physiological measures of bottle feeding and sucking (length of sucking bursts, peak-to-peak intervals, rate of sucking, suck-swallow rations and percentage pressure generation above baseline pressure in the feeding bottle) using the Great Ormond Street Measurement of Infant Feeding (GOSMIF). Finally, there was no significant difference between the intervention and control groups for either type of cleft for the number of abnormal swallowing behaviors as assessed by videofluoroscopy (21 patients). At twelve months of age, 6 months after surgical closure of the soft palate, all infants were rated as having normal oral motor skills using the Schedule of Oral Motor Assessment (SOMA) (21 patients).

**Fig 2:**
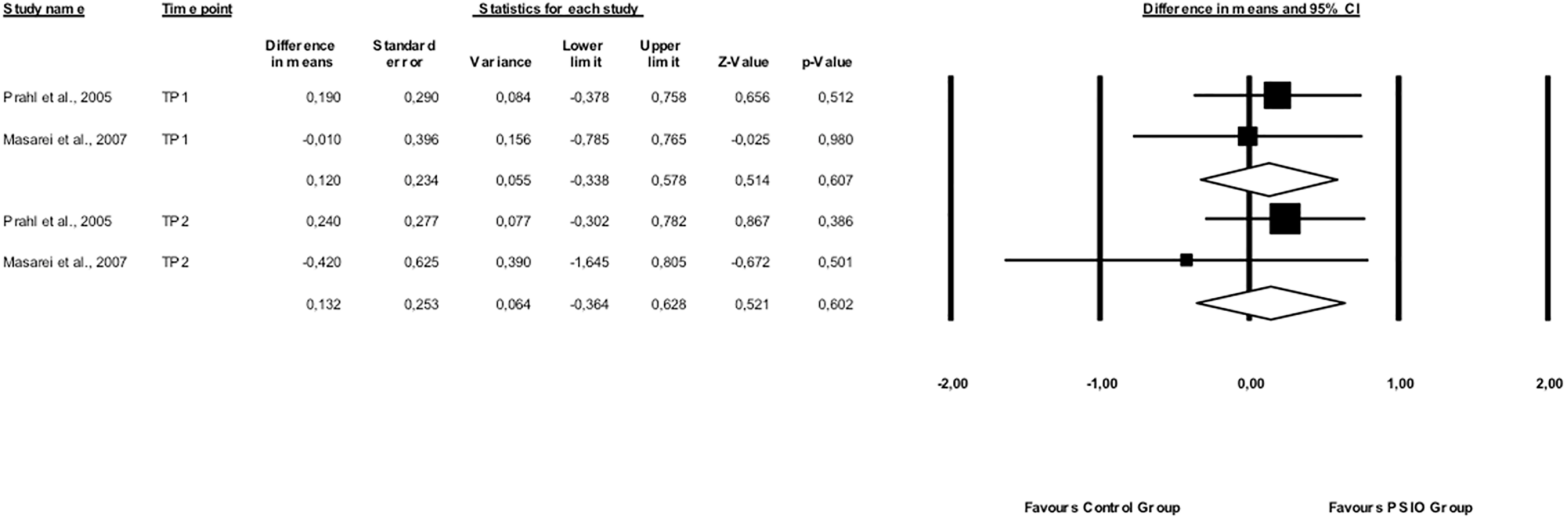
Weight (z scores) of PSIO treated UCLP children compared to control at 3–4 months, before any surgical intervention (TP1: Time Point 1), and at around 12 months of age, after surgical closure of the soft palate (TP2: Time Point 2).

Regarding ***nutritional status***, no statistically significant benefit was observed in weight (z scores) of PSIO treated UCLP children at 3–4 months, before any surgical intervention, [Weighted Mean Difference (WMD): 0.120, (95% Confidence Interval (CI): -0.338–0.578), *p* = 0.607; 2 studies, n = 72; I^2^ = 0%] and at around 12 months of age, after surgical closure of the soft palate [WMD: 0.132, 95% CI: -0.364–0.628, *p* = 0.628; 2 studies, n = 42; I^2^ = 0%] (Fig 2).

**Fig 3:**
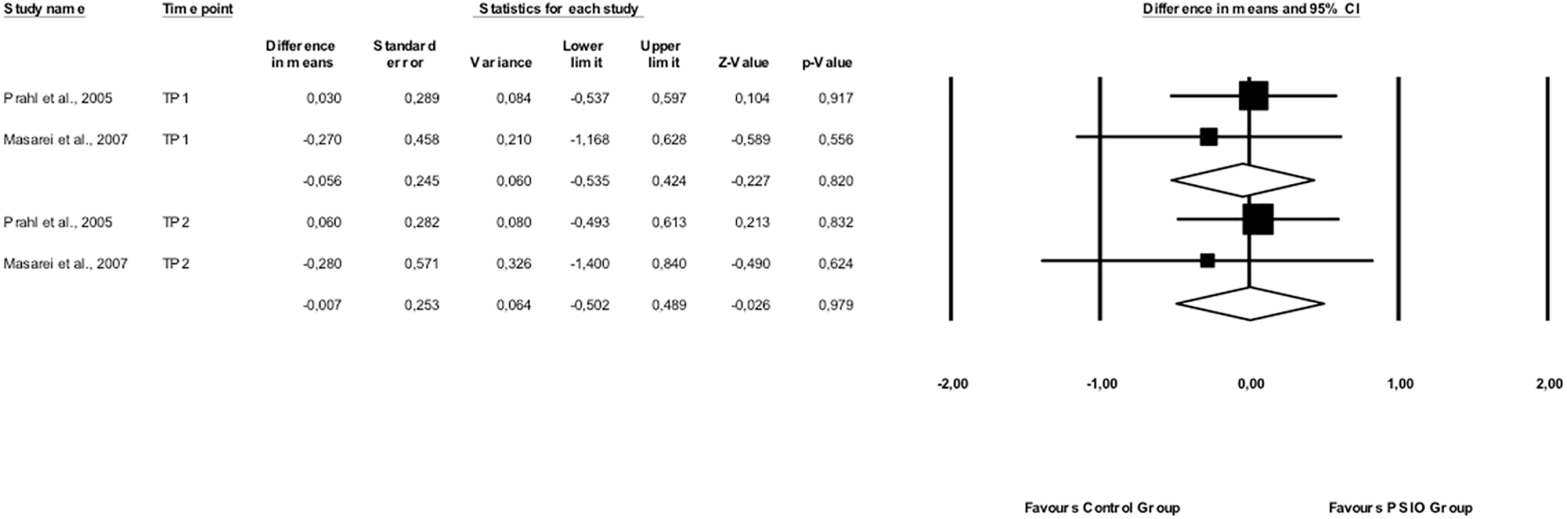
Height (z scores) of PSIO treated UCLP children compared to control at 3–4 months, before any surgical intervention (TP1: Time Point 1), and at around 12 months of age, after surgical closure of the soft palate (TP2: Time Point 2).

Similarly, no statistically significant benefit was observed in height (z scores) of PSIO treated UCLP children at 3–4 months, before any surgical intervention, [WMD: -0.056, 95% CI: -0.535–0.424, *p* = 0.820; 2 studies, n = 72; I^2^ = 0%] and at around 12 months of age, after surgical closure of the soft palate [WMD: -0.007, 95% CI: -0.502–0.489, *p* = 0.979; 2 studies, n = 52; I^2^ = 0%] (Fig 3). Moreover, Prahl et al. [31] observed that the mean z scores for weight-for-length in the UCLP intervention group children were significantly lower after soft plate closure. Similarly, Masarei et al. [8] noted that infants did not differ in head circumference at three months, or in head circumference and Body Mass Index at the twelve-month assessment. Finally, no differences were noted for the isolated cleft palate group, at any time point (16 and 11 patients, respectively).

Based on these two papers [31, 8], the quality of available evidence, using the GRADE approach [20], for the outcomes of weight and height evaluated at approximately 12 month of age was considered as very low (S8 Table).

#### Facial esthetics

Two studies analyzing data from the DUTCHCLEFT sample at various ages discussed the effects of PSIO on facial esthetics [33, 34]. In the first study, 41 subjects of 18 months of age, 21 of them treated with the PSIO appliance, were assessed, using photographs, by professional and adult laypeople judges. Two photographs, one showing the full face and a cropped version focusing on the mouth and nose area, were used to judge facial appearance by comparison to similar versions of a reference photograph (i.e. a good and clear photograph in the middle of the range from poor to excellent aesthetic outcome). The experimental photograph was presented together with the respective reference one and received a comparative score and a position on a Visual Analog Scale (VAS) and these scores were then pooled together. The score for the reference photograph was arbitrarily set a 100 and the assessors were instructed to increase it or decrease for the test view if they believed the esthetic outcome was better or worse respectively. Similarly, the reference photograph was given a specific position on the VAS and the judges were asked to move this position towards the right limit of the scale if they believed the outcome in the test photo was more esthetic compared to the reference one, or the opposite. No statistically significant differences were found between the intervention and the control group for the full face or the cropped photographs (z-scores).

In a subsequent study from the same sample [34], 45 children (24 intervention and 21 control group) were evaluated at the age of 4 and 46 children (22 intervention and 24 control group) at the age of 6 years with a similar methodology. At the age of 4 years, the full-face photos of subjects in the treatment group were judged to be more attractive than photos of children belonging to the control group. However, at 6 years of age this difference persisted only for the photos cropped to focus on the mouth and the nose judged by professionals. Regression analysis showed a minor effect of occlusion on the judgments of the full-face photographs.

The quality of available evidence assessed with the GRADE was considered as low (S9 Table).

#### Dentofacial cephalometric variables

One study analyzing data from the DUTCHCLEFT sample investigated the effects of PSIO on dentofacial cephalometric variables (angular, linear and ratio variable representing soft and hard tissues, as well as, dental structures) [36]. Forty-one children (21 intervention and 20 control group) were evaluated at the age of 4 and 43 children (21 intervention and 22 control group) at the age of 6 years with a similar methodology. At first assessment, the interincisal angle was about 9 degrees larger in the treated group, a difference not verified in the latter evaluation. However, at 6 years of age, the mentolabial angle was almost 9 degrees smaller in the intervention than the control group.

The quality of the available evidence for selected cephalometric measurements assessed with the GRADE approach was considered as low (S10 Table).

#### Maxillary arch dentoalveolar variables

Three publications analyzing data from the DUTCHCLEFT sample investigated the effects of PSIO on maxillary dentoalveolar variables at different ages [5, 28, 32].

Prahl et al. [28] and Bongaarts et al. [32] studied the ***contact and collapse of the alveolar segments*** in maxillary casts at various ages. In the first study, a total of 49 children (24 in the intervention group and 25 in the control) were assessed shortly after birth when comparable arch forms with no contact or overlap of the maxillary segments were observed in both groups. At subsequent assessments at 15 weeks of age (prior to lip repair), 24 weeks (6 weeks after lip repair), 48 weeks (4 weeks prior to soft palate closure), 58 weeks (approximately 6 weeks after soft palate surgery and at 78 weeks (half a year after soft palate closure) the frequency of collapse and its severity increased similarly between the two groups. Bongaarts et al. [32] produced corroborating evidence at the 4 year and 6 year assessments, after examining the casts of 45 children (23 in the intervention group and 22 in the control at the first measurement, and 22 in the intervention group and 23 in the control at the second).

Various ***linear and angular maxillary dentoalveolar variables*** changes were assessed three dimensionally in casts and presented in the papers of Prahl et al. [5] (24 children in the intervention and 25 in the control group; assessed shortly after birth and at 15 weeks (before lip surgery), 24 weeks, 48 weeks, 58 weeks and 78 weeks of age (6 months after soft palate surgery)) and Bongaarts et al. [32] (4 years assessment: 23 children in the intervention and 22 in the control group; 6 years assessment: 22 children in the intervention and 23 in the control group).

At the 78-week examination, the only statistically significant difference, between the two groups in the assessed variables, was observed in the change from the baseline of anterior arch depth. This variable increased more in the intervention group compared to the control children. No other statistically significant change from the baseline was noted in variables regarding cleft width (alveolar cleft width, midpalatal cleft width, posterior cleft width at the tuberosity level), as well as, linear arch dimensions (anterior arch width, tuberosity width, total arch depth, total arch length, alveolar cleft margins length) and angular measurements considered (angulation of the greater alveolar segment in the transverse plane of space, angulation of the smaller alveolar segment in the transverse plane of space, vertical slope of the greater alveolar segment, vertical slope of the smaller alveolar segment).

The comparison of the various maxillary arch variables at 4 years of age revealed a statistically significant increase in total arch depth in the treated group and the angulation of the greater alveolar segment in the transverse plane of space. At the 6-year re-evaluation, no statistically significant difference was observed between the two experimental groups.

The quality of the available evidence for arch width and arch depth was considered as low (S11 Table).

#### Dental arch relationships

The effect of PSIO on dental arch relationships was investigated in two papers analyzing data from the DUTCHCLEFT sample at different time points [29, 38].

Bongaarts et al. [29] evaluated occlusion at 4 years (22 children in the intervention and 22 in the control group) and at 6 years (22 children in the intervention and 23 in the control group). No statistically significant differences were observed regarding the 5-year old index (categorizing arch relationships using reference models), the Huddart/Bodenham score (dental arch relationships at the transverse planes), overjet, overbite and sagittal occlusion assessment (scored for deciduous canines and second deciduous molars according to Angle classification). Corroborating evidence was produced by assessing occlusion with the Huddart/Bodenham score modified for mixed dentition at 9 (24 children in the intervention and 21 in the control group) and 12 (22 children in the intervention and 22 in the control group) years of age [38].

The quality of the available evidence for the total and buccal Huddart/Bodenham score was considered as low (S12 Table).

#### Hearing, speech and language evaluation

The evaluation of different aspects of speech and language development in the DUTCHCLEFT sample of patients started at age 1 and continued at 6-month-intervals until 3 years of age. Language development was also assessed at a 6-year follow-up. Subjects included had both parents fluent in the Dutch language and were first evaluated regarding pre-lexical development (sounds produced in babbling) at age 1 and 1.5 [22]. Subsequently, phonological development of the children was followed from age 2 to 3 [25] and an interim speech quality and intelligibility assessment was performed at age 2.5 [23, 24, 27]. Moreover, during the same time period (from age 2 to 3), patients’ hearing status and language skills development was investigated. Finally, expressive language skills were re-assessed at six years of age [26].

Assessment of ***pre-lexical development*** (Koopmans-van Beinum and Van de Stelt’s system: classification of utterances, phonation characteristics, articulation movement, phonetic inventory) was performed in 36 infants aged 12 months (18 in the intervention and 18 in the control group) and 38 infants aged 18 months (19 in the intervention and 19 in the control group). Development from 12 to 18 months was followed in 31 children (15 in the intervention and 16 in the control group). At 12 months of age, babies using the PSIO appliance showed improved use of alveolar articulations, however, the effects of PSIO seemed to be of a transient nature. At age 1.5 years, changes towards more normal articulation occurred in both groups and no significant differences in all variables of sound production were noted between them [22].

Subsequent ***phonological development*** was investigated with a system specific for Dutch children (Fonologische Analyse van het Netherlands (FAN) [25]. Only six toddlers (4 in the intervention and 2 in the control group) were assessed at all three time points. At the 2-year evaluation, 16 children participated (9 in the intervention and 7 in the control group), at the 2.5-years group, 18 children were assessed (9 in the intervention and 7 in the control group), and at 3 years of age, 12 toddlers (6 in the intervention and 7 in the control group) were evaluated. Subjects from the intervention group exhibited a more normal path of phonological development during the observation period. At the 2.5 years evaluation, this was classified as normal or delayed, whereas most control toddlers were categorized as belonging to the abnormal developmental pattern. In addition, at the age 3 assessments, children of the former group had acquired more initial consonants than those in the non-treated one. No other statistically significant differences were observed in the use of phonological processes or the occurrence of nasal escape.

During the same chronological age interval, the ***speech quality*** of 10 toddlers 2.5 years-old (10 in the intervention and 10 in the control group) was evaluated perceptually (on seven-point equal-appearing interval rating scales) regarding 13 specific characteristics together with an assessment of an overall impression of speech quality [27] and of the need for speech therapy in the year following the experiment [24]. Based on the results of this study, children belonging to the intervention group obtained significantly higher ratings for intelligibility than the control group children. This result was in agreement with the finding of an earlier study (10 toddlers 2.5 years-old; 10 in the intervention and 10 in the control group), which had assessed speech intelligibility by means of rating on a ten-point rating scale marked by the contrasting labels ‘unintelligible’ (rating 1) to ‘intelligible’ (rating 10) [23]. However, these ratings did not completely reflect intelligibility defined as the proportion of words understood by the listener, as data obtained by means of transcriptions indicated that, in fact, there were no group differences in actual intelligibility.

The evaluation of different aspects of speech and language in the DUTCHCLEFT sample was complemented by assessment of patients’ ***language skills development*** [26]. At the age of 2, 2.5, and 3 years linguistic development was evaluated in a group of 12 toddlers (6 in the intervention and 6 in the control group). Receptive language skills were investigated using the Dutch version of the Reynell Developmental Language Scales and expressive language skills by calculating the variables mean length of utterance (MLU) and mean length of longest utterances (MLLU). No differences in receptive language skills were noted, however, at age 2.5 and 3 years, the toddlers belonging to the treated group performed better regarding expressive language skills. In the fraction of these patients (6 in the intervention and 5 in the control group), which were re-evaluated at 6 years of age using standardized Dutch language tests, the difference in expressive language between the experimental groups ceased to be significant.

Evaluation of the ***hearing status*** in the context of the latter study [26] revealed that hearing thresholds and presence of middle early infection did not present gross differences between the two groups at the age of 2, 2.5, and 3 years.

The quality of the available evidence for speech intelligibility at 2.5 year-old toddlers was considered as very low (S13 Table).

#### Patient and caregiver-reported outcomes

One publication presenting data from the DUTCHCLEFT sample investigated the effects of PSIO on caregiver-reported outcomes [35].

In total, the mothers of 54 infants were asked to complete and return a questionnaire when their children were 6, 24 and 58 weeks old. The questionnaire contained 42 questions, completed on a 4-point scale (from 1—very satisfactory, very happy, a lot of fun, more than adequate—to 4—very unsatisfactory, very unhappy, no fun, very inadequate) and categorized in the domains of interaction and caretaking of the baby, comings and goings of the baby, motherhood and life outside and support.

Statistical analysis of the questionnaires finally provided by 49 mothers did not locate any significant differences between the two groups. The quality of the available evidence for the mean satisfaction scores of the derived from the questionnaire as a whole (four domains) was considered as low (S14 Table).

#### Economic evaluation related outcomes

Three papers eligible for inclusion in the present thesis gave comparison data on economic evaluation related outcomes, two of them from the DUTCHCLEFT sample [8, 21, 30].

Regarding analysis of the ***costs associated with PSIO appliance treatment and calculations on cost-effectiveness***, until surgical closure of the lip (around 18 weeks of age) total direct medical costs (calculated on the cost of personnel, cost of PSIO materials and overhead cost) expressed in US$ (1994 base year) in the intervention group were significantly more (852 ±69 US$ for 23 infants) that in the control group (304 ±34 US$ for 20 infants). The same was observed for indirect non-medical costs (calculated on the number of visits, the number of parents that would normally accompany the child, the average time of the visit, the duration of travel, and the normal daily activity of the accompanying parent (s)) expressed in 1994 base year US$ (231 ±106 US$ for 15 infants in the intervention group and 130 ±14 US$ for 14 infants in the control group). However, no statistically significant difference was observed between two groups with regards to the duration of surgical lip closure (23 children in the intervention and 20 children in the control group) [21].

For the whole duration of PSIO appliance treatment, the costs submitted by the orthodontist in € (2002 base year) were 1460 ±247 € for 10 infants in the intervention and 419 ±91 € for 10 infants in the control group (Konst et al., 2004) [30]. The calculated cost effectiveness from the perspective of speech development (total impression of speech quality at the age of 2.5 years) was 1041 € for 1.34 points of speech quality improvement.

The quality of the available evidence for the cost of treatment by the orthodontist in the period from birth to soft palate closure was considered as very low (S15 Table).

Regarding the ***visits to the orthodontist***, for the first 18 weeks, 7.2 ±1.8 were needed for the 23 infants in the intervention and 2.4 ±0.6 € for 20 infants in the control group [21]. For the whole period of PSIO appliance intervention, statistical significantly more visits were reported for the PSIO group, both in the Prahl et al. study [5] (for the 12 month duration of PSIO treatment; mean difference: 9.4; 95% CI: 8.02–10.78) and the Masarei et al. study [8] (for the 6 month duration of PSIO treatment; mean difference: 2.68; 95% CI: 0.83–4.53)

#### Adverse effects and problems related to PSIO appliances and procedures

Possible adverse effects and problems related to PSIO appliances and procedures were not systematically investigated in the studies included in the present review comparing the effect of PSIO appliances to no treatment.

Only the Masarei et al. [8] paper reported on possible problems related to PSIO appliances and procedures. In approximately 20 percent of infants (9 out of 50) the operators reported loose fitting plates, which were corrected. Other minor problems included oral thrush, minor ulceration and neonatal teeth.

### Comparative effect of PSIO procedures

Only one study [37] compared the effects between two PSIO procedures. No difference was noted for *nostril height*, *nostril sill height* and *nostril area ratio* at any available time point of measurement. Only for the variable nostril width ratio was an increase observed after PSIO procedures with the Figueroa technique. However, six months after surgical correction this difference had ceased to exist. The quality of the available evidence for nostril height and nostril width ratios was considered as low (S16 Table).

Regarding analysis of the *costs associated with PSIO appliance treatment*, no statistically significant difference was noted with regards to total costs of treatment for parents/caregiver (Figueroa group 1240 ±250.14 US$; Grayson group 1159 ±275.81 US$; *p* = 0.357) and total cost of treatment for national insurance (Figueroa group 20267 ±1668 US$; Grayson group 19667 ±1839 US$; *p* = 0.357). The quality of the available evidence for the costs associated with PSIO appliance treatment was considered as low (S17 Table).

Regarding the *visits to the orthodontist(s)*, no statistically significant difference was observed regarding the total number of visits before surgical correction (Figueroa group 8.33 ±1.59; Grayson group 7.67 ±1.84; *p* = 0.297).

Regarding *adverse effects and problems related to PSIO appliances and procedures*, only a statistically significantly increased occurrence of alveolar ulceration in the Grayson group was noted. No other differences were observed regarding nasal ulceration, skin rash or superficial skin injury caused by taping.

### Risk of bias across studies

Due to the insufficient number of trials identified for each outcome, it was not possible to conduct subgroup analyses, or analyses fro “small-study effects” and publication bias [15].

## Discussion

### Summary of evidence

PSIO concept has been integrated into the standards of care for cleft lip and/or palate patients in many dedicated treatment teams around the world [5–8]. However, based on the data retrieved in the present systematic review, the investigated appliances did not present significant effects when compared to each other or to no treatment.

From the initially identified records, twenty full-text reports from randomized controlled trials on children with unilateral cleft lip and palate, as well as, cleft palate were included in the systematic review. No studies on children with bilateral cleft lip and palate were retrieved. Eighteen of the eligible publications reported on various outcomes originating from the sample of a single study. In essence, only three randomized controlled trials satisfied the pre-specified criteria for inclusion in the present paper, reflecting the scarcity of relevant research at the top of the widely accepted hierarchy of scientific evidence. Considerable research has been done in trials that were not randomized despite the fact that it is widely accepted that well-designed and properly executed RCTs provide the best evidence on the efficacy of health care interventions [39, 40]. Among these three acceptable RCTs, only one (Dutchcleft) followed patients from approximately 2 weeks after birth until 12 years of age. The consequent lack of extensive data of high evidence based potential is rather surprising bearing in mind not only the prevalence of the problem [1], but also the fact that its manifestations are usually life-long and associated with significant morbidity [41]. Thus, relevant, evidence-based information would be beneficial in order to support the care provided for these children with possible consequences to a broad spectrum of human life, like growth and development, esthetics, speech and language development, as well as, various psychosocial parameters.

#### Effect of pre-surgical infant orthopedics vs. no treatment

In general, based on the information provided from the two study samples eligible for inclusion in the present review, PSIO does not exhibit clinically significant effects over no treatment. Two papers [8, 31] included outcomes related to *feeding and general body growth*; functions and outcomes considered of the utmost importance in evaluating the early stages of human life. According to the results of the two studies, which were consistent with each other, neither passive nor active PSIO appliances, have discernible positive effects on feeding functioning and the subsequent nutritional status in patients with unilateral cleft lip and palate or isolated cleft palate. A recent systematic review of randomized controlled trials focusing exclusively on growth and development outcomes assessment after various feeding interventions in infants with cleft lip and/or palate reported similar findings [13].

Furthermore, passive PSIO appliances did not generally exert a significant influence on *facial esthetic perception*, which constitutes another important concern related to this condition [33, 34]. At the longest follow-up at 6 years of age, the PSIO treated group got better ratings when professionals assessed photographs focusing on the nasolabial region. Judgments on cropped photographs are considered more reliable because they may help blind other parameters, such as general characteristics of the baby face and variation in the response to facial expressions that have been found to positively affect overall ratings [42–46]. However, this beneficial effect observed in professional ratings was considered irrelevant in daily life, where affected children interact with ordinary people.

Cephalometric investigation of *facial growth* in the Dutchcleft sample did not reveal any clinically relevant effect of passive PSIO appliances either [36], although it is not totally clear whether their results could have been influenced by the uncertainty inherent in landmark localization [47–50]. In addition to common errors in cephalometric investigations, the presence of malpositioned incisors in an atrophic and displaced premaxilla could be regarded as an additional cause of measurement errors in young cleft patients [34, 51]. In the case of unilateral cleft lip and palate toddlers, alternatives for cephalometric radiographics points A, ANS, and PNS have been assessed but not proven to perform better than traditional landmarks [52]. Overall, the level of measurement errors in the Bongaarts et al study [36] was considered acceptable.

Investigation of orofacial region growth by means of study casts also failed to reveal any discernible effects of passive PSIO appliances at the longest follow-ups available. PSIO processes were not able to prevent collapse of the maxillary arch or influence maxillary arch dimensions in 6-year-old children [32]. It may be worth noting that non-RCTs have produced data on significant differences for some of the maxillary arch measurements in younger children [53–56].

Moreover, assessment of occlusal relationships using the modification of the Huddart/Bodenham system showed no differences between treatment and control groups at 12 years of age [38], which conforms to the results of a non-RCT evaluating the effect of an active PSIO appliance with the GOSLON yardstick in younger children [57]. This modification of the Huddart/Bodenham system, which is used to examine dental arch relationships in the transverse place of space, is regarded to be a powerful, valid and reliable measurement of treatment outcome in cleft lip and palate children [58–60].

In general, when considering overall growth in cleft lip and palate patients, it should be noted that since individualization of the treatment is expected, the type and frequency of the various therapeutic interventions may constitute confounding factors; something that remains to be investigated in depth [38]. Moreover, growth and treatment parameters may not be the only factors affecting the general esthetic perception in patients with cleft lip and palate, a subject of particular and life-long importance [34, 36]. Additional parameters, including but not limited to facial expression, skin texture, hair and eye color, may exert greater influence than expected, effects needing to be studied at long follow-ups as they may become more prominent following the pubertal growth [34].

Regarding *speech and language development*, 2.5 year-old toddlers belonging to the intervention group not only obtained significantly higher ratings for intelligibility than the control group children [23], but these scores were high enough for the whole intervention to be considered cost-effective from a societal economic evaluation point of view [30]. However, these results should be considered with caution; firstly because these ratings did not completely reflect intelligibility defined as the proportion of words understood by the listener [23] and secondly because no sound longer-term data on the broader subject exist. Only the data from a preliminary assessment of 12 patients’ language skills development at 6 years of age suggested that there was no difference between treated and untreated groups [26]. However, a similar lack of difference between the two groups was also noted in two relevant non-RCTs [7, 61].

Finally, no statistically significant outcomes were observed regarding caregiver *psychosocial related outcomes* like satisfaction in motherhood [35]. However, these results were obtained using an instrument not previously tested and validated regarding its psychometric properties.

#### Comparative effect of PSIO procedures

No statistically important differences were observed between the modified Figueroa and the modified Grayson nasoalveolar molding techniques regarding nasal *facial esthetics* six months after surgical correction of the lip [37]. Even though some studies have indicated an advantageous use of pre-surgical nasoalveolar molding for the improvement of nasal symmetry [62–67], these results are not universal [68] and remain to be supported by RCTs.

Both approaches, from an *economic evaluation* approach, incurred significant costs for parents/caregivers and national insurance institutions. The only difference noted was in *adverse effects and problems related to PSIO appliances and procedures*, namely a statistically significant increased occurrence of alveolar ulceration in the Grayson group.

#### Quality of the available evidence

Overall, the quality of evidence assessed with the GRADE approach [20] was considered at best as low, indicating caution regarding the strength of the relevant recommendations.

Available information on the vast majority of outcomes considered was based on data from one randomized controlled trial, namely the Dutchcleft study sample, indicating the *scarcity of evidence based information* on a problem with such significant and life-long consequences for both patients and their families. Only regarding general body growth outcomes during the first year of life was the literature search able to retrieve data from two separate data sets and an exploratory quantitative data synthesis was attempted. The results obtained from these studies were similar and the I^2^ statistic obtained from the meta-analytic calculations suggested a relatively unimportant degree of heterogeneity, indicating that *inconsistency* during the GRADE assessment was not to be considered serious. In the context of the present review heterogeneity can arise from diversity in terms of the characteristics of patients and interventions, as well as measurement methodology related factors and was incorporated into a justifiable random effects model.

An important factor leading to downgrading the overall quality of evidence originated from the *risk of bias assessment* for the outcomes considered in the included studies. Overall, outcomes included in eight of the retrieved publications studies were considered as being of low risk of bias [5, 28, 29, 32–34, 36, 37] and four of unclear [8, 31, 35, 38]. The outcomes included in eight publications, regarding speech and language development and economic evaluation outcomes, were considered as being of high risk of bias [21–27, 30]. As all studies included in the present review were considered to present low risk of bias regarding the domains of random sequence generation, allocation concealment, blinding of the participants, caregivers and the personnel and blinding of outcome assessment, the examination of the rest of the domains considered produced results that contributed to the variable overall assessment.

Many problems regarding risk of bias were attributable to incomplete outcome data. In two out of three patient samples included in the present review, patients finally analyzed were less than those originally randomized (Ducthcleft sample) or those initially calculated during power sample calculations [8]. Of course, it is absolutely expectable that in randomized controlled trials, especially those evaluating outcomes after lengthy time periods, a significant proportion of participants may be not evaluated for various reasons. Especially in the Ducthcleft study, an attempt to repeat the power calculations (based on SNA difference at 4 years of age) during study progression for some of the variables (SNA, ANB, 5-year index, esthetic score) [36] was made and it was then considered that intervention and control groups were large enough to show significant differences, if any were present. However, these calculations may not be applicable for other outcomes. Moreover, *post hoc* power calculations, with the G*Power software (version 3.1.9.2) [69, 70], using the actual results reported showed that the achieved power in most instances failed by a wide margin to achieve the initial 80% setting. Thus, in some cases the risk of bias from missing data was considered unclear while in others, especially those evaluating speech and language development and economic evaluation outcomes, the risk of bias regarding this domain and overall was considered high.

Some assessments of outcomes included in the material retrieved, were considered to present other threats to validity. For example, in some cases, no definite conclusions could be reached regarding the effect of varying compliance with PSIO appliances wear could have on study results. All included publications reported some relevant data, most probably derived from questioning the parents or the caregivers. However, this method is considered to be less effective despite its frequent use [71]. In addition, in many instances the effect of various surgical or orthodontic interventions, supplementary to the original protocol, especially in the papers reporting data on the longer follow-ups was not clear-cut, thus creating uncertainties regarding possible bias in the retrieved data.

In all the cases considered, the overall quality of evidence was downgraded because of problems related to serious *indirectness* of the evidence retrieved and problems related to *imprecision*. The results obtained were derived from specific populations (particular ethnic background and cleft type) and treatment protocols; hence even this limited set of data cannot be applied with certainty in clinical settings characterized by a different patient mix or variable type and frequency of treatment modalities. Moreover, for varying reasons, the numbers of patients analyzed were limited, creating serious problems regarding the precision of the results obtained.

### Strengths and limitations

The strengths of the present review include using a methodology following well-established guidelines and the fact that it focused exclusively on randomized controlled trials, as it is widely accepted that well-designed and properly executed RCTs provide the best evidence, with decreased risk of bias, on the efficacy of health care interventions [39, 40]. The available empirical evidence suggests that intervention effects in orthodontic research seem to differ in non-RCTs compared to RCTs [72]. The two systematic reviews published recently that evaluated PSIO appliances in general [11, 12], also included non-RCTs. Furthermore, neither attempted to summarize the quality of available evidence and thus provide an insight into the strength of the relevant recommendations, as was carried out in the present systematic review based in the GRADE approach [20].

Moreover, the search strategy employed in the present review was both exhaustive, covering electronic, manual, and gray literature material up to May 2016, and comprehensive including every available randomized controlled trial making comparisons between PSIO clinical applications, or to no treatment, irrespective of language, date and status of publication. Every effort to decrease bias in the methodology employed was made. Screening, verification of eligibility, abstraction of information, assessment of risk of bias and of the quality of evidence were performed in duplicate, and any disagreement was resolved by discussion or consultation with the thesis co-supervisor until a final consensus was achieved. Finally, the random effects model was employed during exploratory quantitative data synthesis to incorporate any observed heterogeneity [73].

There are also some limitations to the present review, arising mainly from the nature and the characteristics of the data retrieved during the review process, which resulted in the assessment of the level of available evidence as, at best, low. The scarcity of relevant high quality hierarchically evidence based information from RCTs, precluded meta-analytic procedures for most outcomes. Even in cases where such an approach was attempted, such quantitative syntheses can only be regarded as exploratory until additional research becomes available. However, current concepts support that data from even as few as two studies can be combined, provided that these can be meaningfully pooled [74], as all other summarizing techniques are less transparent and/or are less likely to be valid [75]. Furthermore, exploratory subgroup analyses and analyses for “small-study effects” and publication bias [15], could not be carried out even though they were incorporated as possibilities according to the review protocol.

Another limitation of the data retrieved in this study stems from the small number of patients finally analyzed resulting an increased risk of bias and subsequent problems regarding the precision of the effect estimates in some cases. Moreover, it has to be acknowledged that the results of this review relate mostly to the comparison of the passive PSIO appliances to no treatment in Dutch infants with unilateral cleft lip and palate, since this constitutes the overwhelming majority of the information obtained. The inclusion criteria applied in the Dutchcleft study precludes the application of the results to other populations and procedures, thus diminishing the directness and generalizability of the available evidence. No studies on children with bilateral cleft lip and palate were retrieved. In addition, very limited data was locatable regarding active PSIO appliances or nasolaveloar molding techniques.

There was an additional limitation of the material included in this study originating from supplementary interventions carried out in some patients as part of the unavoidable individualization of treatment protocols required to cater for individual patient needs. However, the influence of these confounding parameters could not be clarified, either in the individual studies included in the present review, or as a part of subgroup analysis because of the lack of extensive relevant data. Finally, the involvement of different operators is also to be expected in long-term evaluations and can form a source of variability. This issue may constitute an additional reason of cautiousness when interpreting the results of this review.

### Recommendations for future research

Evaluation of treatment modalities in the area of craniofacial abnormalities should be a continuous activity with use of proper scientific methodology [76]. As the overall quality of the relevant available evidence was considered at best as low, further research is imperative in order to discern more specifically whether various PSIO appliances and treatment protocols exert or not any justifiable effect on different parameters in the long term. This would allow orthodontists to reach specific and robust recommendations useful in the clinical setting. Well-designed and properly executed RCTs provide the best evidence with decreased risk of bias on the efficacy of health care interventions [39, 40]. Long-term evaluation of the outcomes of different treatment protocols is extremely valuable, since it is well known and understood that the definitive treatment outcomes in patients with cleft lip and/or palate cannot be recognized until facial development is complete [77].

To facilitate any type of research project thorough and proper records should be obtained from every patient, including traditionally orthodontics casts, facial photographs and radiographs. Recently, the use of three-dimensional records has been advocated. Also, a general agreement should be reached regarding the timing and type of records to be used. The EUROCRAN project (www.eurocran.org), advocates a fixed protocol regarding type and timing of records obtained. With a standardized set of records, collaboration and follow up can be simplified, while the inter-study comparison of outcomes may be facilitated by the use of meta-analytic techniques [78].

Equally important, consensus has to be reached on the outcome measures related to psychological variables and quality of life. Clinical trials are only as credible as their outcomes [79]. A patient-centered approach, incorporating assessment of outcomes like patient satisfaction and the quality of life is a crucial part of treatment outcome and quality management and therefore it should be included in any relevant study [80].

Furthermore, the vast majority of evidence-based data up to the present have involved a specific passive PSIO appliance protocol used in a particular Western European population with cleft lip and palate. Future research should also be directed to alternative PSIO protocols, as well as, populations of different ethnic origin and patients with bilateral cleft lip and palate, so as better understanding of the different aspects of treatment can be achieved. In addition, every effort should be made to increase the number of patients recruited, retained and analyzed in such studies, although the associated problems are well comprehended and expected. Increasing the number of patients analyzed will diminish the chance of a Type II error; at the same time it will increase the precision of effect estimates [78]. This will, in addition, facilitate the investigation of the effect of the confounding factors of individualized treatment interventions on the overall effect of PSIO appliances.

Finally, as resources are always limited in the context of health-care systems, investigation of the cost-effectiveness of the various treatment approaches is imperative [21] as is also the examination of possible adverse effects and problems related to PSIO appliances and procedures in order to enhance the quality of the delivered care [81].

## Conclusions

Based on the findings of the present systematic review and meta-analysis, conducted following well-established guidelines, the null-hypotheses are not rejected. It seems that the investigated PSIO protocols used in patients with cleft lip and/or palate, generally, do not present significant effects when compared to each other or to no treatment, in terms of feeding characteristics and general body growth, facial esthetics, cephalometric variables, maxillary dentoalveolar variables and dental arch relationships, speech and language related variables, caregiver-reported outcomes, economic evaluation related outcomes, as well as, adverse effects and problems related to the appliances or the applied procedures. The aforementioned findings could provide initial guidance in the clinical setting, bearing in mind the small number of eligible trials, their heterogeneity with regards to treatment protocols, the results of the risk of bias assessment, as well as, the overall low quality of the available evidence. Given the multitude of parameters, which may have affected the results of the included trials, good practice would suggest further research in the respective field, in order to increase the body of high quality evidence and reach more robust relevant recommendations for management decisions in individual cases.

## Supporting information

S1 Checklist PRISMA checklist. (DOC)

S1 File PROSPERO protocol. (DOCX)

S1 Table Strategy for database search [until May 1^st^, 2016]. (DOCX)

S2 Table General characteristics of the studies included in the systematic review–Publications from the DUTCHCLEFT. (DOCX)

S3 Table General characteristics of the studies included in the systematic review–Remaining studies. (DOCX)

S4 Table Participant characteristics of the studies included in the systematic review–Publications from the DUTCHCLEFT. (DOCX)

S5 Table Participant characteristics of the studies included in the systematic review–Remaining studies. (DOCX)

S6 Table Details of risk of bias assessment–Publications from the DUTCHCLEFT. (DOCX)

S7 Table Details of risk of bias assessment–Remaining studies. (DOCX)

S8 Table Quality of available evidence for the outcomes of weight and height. (DOCX)

S9 Table Quality of available evidence for facial esthetics assessment. (DOCX)

S10 Table Quality of available evidence for selected cephalometric measurements. (DOCX)

S11 Table Quality of available evidence for selected maxillary arch dentoalveolar variables. (DOCX)

S12 Table Quality of available evidence for Huddart/Bodenham scores. (DOCX)

S13 Table Quality of available evidence for speech intelligibility. (DOCX)

S14 Table Quality of available evidence for total questionnaire scores. (DOCX)

S15 Table Quality of available evidence for total cost of treatment by the orthodontist. (DOCX)

S16 Table Quality of available evidence for nostril height and nostril width ratios. (DOCX)

S17 Table Quality of available evidence for cost comparison between different nasoalveolar molding techniques. (DOCX)
