# Supplementary material for: Treatment outcomes of pre-surgical infant orthopedics in patients with non-syndromic cleft lip and/or palate: A systematic review and meta-analysis of randomized controlled trials
Source: PLoS One. 2017 Jul 24;12(7):e0181768. doi: 10.1371/journal.pone.0181768 (PMC5524403; doi:10.1371/journal.pone.0181768)
Supplement: S1 File — (DOCX) [file pone.0181768.s002.docx]

**Hamid Reza Hosseini, Eleftherios G. Kaklamanos, Athanasios E. Athanasiou. Treatment outcomes of pre-surgical infant orthopedic appliances in patients with non-syndromic cleft lip and/or palate: a systematic review and meta-analysis of randomized controlled trials. (PROSPERO 2016: CRD42016047940)**

**Review question(s)**

The aim of this study is to investigate the effectiveness of pre-surgical infant orthopedic (PSIO) appliances in patients with non-syndromic cleft lip and/or palate and evaluate the quality of the available evidence.

**Searches**

Comprehensive electronic database searches will be undertaken without language restriction in the following databases: MEDLINE via PubMed, the Cochrane Central Register of Controlled Trials (CENTRAL), Scopus, Web of Science, LILACS, IndMed, Scielo and Arab World Research Source. Unpublished literature will be accessed electronically using Google Scholar (https://scholar.google.com), ClinicalTrials.gov (http://clinicaltrials.gov), International Standard Randomised Controlled Trial Number (ISRCTN) registry (http://www.isrctn.com) and OpenGrey (http://www.opengrey.eu). In addition, Pro-Quest Dissertation and Theses Global database will be searched. Efforts will be made to obtain conference proceedings and abstracts where possible. Authors will be contacted to identify unpublished or ongoing clinical trials and to clarify methodology and data as necessary. Reference lists of included studies will be screened for additional relevant research.

**Types of study to be included**

The trials to be included should be RCTs evaluating outcomes of PSIO appliance treatment.

**Condition or domain being studied**

Patients with non-syndromic cleft lip and/or palate.

**Participants/ population**

Children of any age with any kind of non-syndromic cleft lip and/or palate defect.

**Intervention(s), exposure(s)**

Any type of PSIO appliance protocol.

**Comparator(s)/ control**

No treatment or alternative PSIO appliance protocol.

**Outcome(s)**

**Primary outcomes**

Feeding characteristics and nutritional status, facial esthetics, dentofacial cephalometric variables, maxillary dentoalveolar variables, dental arch relationships, hearing, speech and language evaluation.

**Secondary outcomes**

Patient and caregiver-reported outcomes, economic evaluation related outcomes, adverse effects and problems related to PSIO appliances and procedures.

**Data extraction, (selection and coding)**

All assessments including titles and/or abstract screening, full text evaluation, and extraction of data will be performed independently and in duplicate by two investigators (HRH and EGK). The investigators will not be blinded to the authors or the results of the research. Disagreements will be resolved by discussion and consultation with a third author where necessary (AEA).

**Risk of bias (quality) assessment**

Assessment of risk of bias will be performed independently and in duplicate by two investigators (HRH and EGK) using the Cochrane Collaboration risk of bias tool that considers seven domains: random sequence generation; allocation concealment; blinding of participants and personnel; blinding of assessors; incomplete outcome data; selective reporting of outcomes; and other potential sources of bias. Each domain will receive a judgement of low, high or unclear risk of bias (indicating either lack of sufficient information to make a judgement or uncertainty over the risk of bias). Studies will be finally grouped into the following categories:

• low risk of bias (plausible bias unlikely to seriously alter the results): if all key domains of the study are at low risk of bias,

• unclear risk of bias (bias that raises some doubt about the results): if one or more key domains of the study are unclear, and,

• high risk of bias (bias that seriously weakens confidence in the results): if one or more key domains are at high risk of bias.

Disagreements will be resolved by discussion and consultation with a third author where necessary (AEA).

**Strategy for data synthesis**

Where studies have used the same type of intervention with the same outcome measure, we will pool the results using a random-effects meta-analysis analysis in view of the likely variation in population groups and settings. Depending on the variation of the indices used to quantify primary or secondary outcomes we will use weighted or standardized mean differences for continuous outcomes and risk ratios for binary outcomes, and calculate 95% confidence intervals and two sided p values for each outcome. Heterogeneity will be assessed using both the Chi-square test and the I-squared statistic.

**Analysis of subgroups or subsets**

If possible studies will be divided into categories depending on participants and intervention characteristics. If an adequate number of trials are identified, we will carry out analyses for “small-study effects” and publication bias.

**Dissemination plans**

Publication in peer reviewed journals.
