# Supplementary material for: Treatment outcomes of pre-surgical infant orthopedics in patients with non-syndromic cleft lip and/or palate: A systematic review and meta-analysis of randomized controlled trials
Source: PLoS One. 2017 Jul 24;12(7):e0181768. doi: 10.1371/journal.pone.0181768 (PMC5524403; doi:10.1371/journal.pone.0181768)
Supplement: S1 Table — (DOCX) [file pone.0181768.s003.docx]

**S1 Table. Strategy for database search [until May 1^st^, 2016].**

| **Database** | **Search strategy** | **Hits** |
| --- | --- | --- |
| **General Sources** |  |  |
| **MEDLINE via PubMed**  http://www.ncbi.nlm.nih.gov/pubmed | (((randomized controlled trial[pt]) OR (controlled clinical trial[pt]) OR (randomized[tiab]) OR (placebo[tiab]) OR (drug therapy[sh]) OR (randomly[tiab]) OR (trial[tiab]) OR groups[tiab])) NOT ((animals[mh] NOT humans[mh])) AND ((cleft lip) OR (cleft-lip) OR (cleft lip and palate) OR (cleft-lip and palate) OR (alveolar cleft*)) AND (infant OR presurgical OR pre-surgical OR preoperative OR pre-operative OR early) AND (orthodon* OR orthopedic* OR orthopaedic* OR nasoalveolar OR moulding OR molding OR pnam OR nam OR plate*) | **173** |
| **Cochrane Central Register of Controlled Trials**  http://onlinelibrary.wiley.com/cochranelibrary | ((cleft lip) OR (cleft-lip) OR (cleft lip and palate) OR (cleft-lip and palate) OR (alveolar cleft*)) AND (infant OR presurgical OR pre-surgical OR preoperative OR pre-operative OR early) AND (orthodon* OR orthopedic* OR orthopaedic* OR nasoalveolar OR moulding OR molding OR pnam OR nam OR plate*) in Title, Abstract, Keywords in Trials | **46** |
| **Scopus**  https://www.scopus.com/ | ((randomized controlled trial) OR (controlled clinical trial) OR randomized OR placebo OR drug therapy OR randomly OR trial OR groups) AND ((cleft lip) OR (cleft-lip) OR (cleft lip AND palate) OR (cleft-lip AND palate) OR (alveolar cleft*)) AND (infant OR presurgical OR pre-surgical OR preoperative OR pre-operative OR early) AND (orthodon* OR orthopedic* OR orthopaedic* OR nasoalveolar OR moulding OR molding OR pnam OR nam OR plate*) AND (LIMIT-TO (SUBJAREA, "DENT")) AND (LIMIT-TO (EXACTKEYWORD , "Human") OR LIMIT-TO (EXACTKEYWORD , "Humans")) | **345** |
| **Web of Science™ Core Collection**  http://apps.webofknowledge.com/ | **TOPIC:** (((randomized controlled trial) OR (controlled clinical trial) OR randomized OR placebo OR drug therapy OR randomly OR trial OR groups) AND ((cleft lip) OR (cleft-lip) OR (cleft lip AND palate) OR (cleft-lip AND palate) OR (alveolar cleft*)) AND (infant OR presurgical OR pre-surgical OR preoperative OR pre-operative OR early) AND (orthodon* OR orthopedic* OR orthopaedic* OR nasoalveolar OR moulding OR molding OR pnam OR nam OR plate*))  **Refined by:** **RESEARCH AREAS:** ( DENTISTRY ORAL SURGERY MEDICINE )  **Timespan:** All years. Search language=Auto | **140** |
| **Regional sources** |  |  |
| **LILACS**  http://lilacs.bvsalud.org/en/ | tw:(orthodon*) AND (instance:"regional") AND ( db:("LILACS") AND type_of_study:("clinical_trials") AND limit:("humans")) | **85** |
| **IndMed**  http://indmed.nic.in/indmed.html | orthodontic AND cleft | **10** |
| **Arab World Research Source [2016 05 01]**  http://0-search.ebscohost.com.amclb.iii.com | orthodont* AND cleft* | **6** |

**S1 Table. Strategy for database search [until May 1^st^, 2016]. [Continued]**

| **Database** | **Search strategy** | **Hits** |
| --- | --- | --- |
| **Grey literature sources** |  |  |
| **Google Scholar**  https://scholar.google.com | allintitle: randomized cleft  Excluding patents & citations | **36** |
| **ClinicalTrials.gov**  http://clinicaltrials.gov/ | cleft lip \| Studies With Results \| Interventional Studies | **2** |
| **ISRCTN registry**  http://www.isrctn.com | ((randomized controlled trial) OR (controlled clinical trial) OR randomized OR placebo OR drug therapy OR randomly OR trial OR groups) AND ((cleft lip) OR (cleft-lip) OR (cleft lip AND palate) OR (cleft-lip AND palate) OR (alveolar cleft) OR (alveolar clefts)) AND (infant OR presurgical OR pre-surgical OR preoperative OR pre-operative OR early) AND (orthodontic OR orthodontics OR orthopedic OR orthopedics OR orthopaedic OR Orthopaedics OR nasoalveolar OR moulding OR molding OR pnam OR nam OR plate OR plates) | **4** |
| **OpenGrey**  http://www.opengrey.eu/ | ((randomized controlled trial) OR (controlled clinical trial) OR randomized OR placebo OR drug therapy OR randomly OR trial OR groups) AND ((cleft lip) OR (cleft-lip) OR (cleft lip AND palate) OR (cleft-lip AND palate) OR (alveolar cleft*)) AND (infant OR presurgical OR pre-surgical OR preoperative OR pre-operative OR early) AND (orthodon* OR orthopedic* OR orthopaedic* OR nasoalveolar OR moulding OR molding OR pnam OR nam OR plate*) | **0** |
| **ProQuest Dissertations and Theses Global**  http://search.proquest.com | ti((cleft lip) OR (cleft-lip) OR (cleft lip and palate) OR (cleft-lip and palate) OR (alveolar cleft*)) | **184** |
