# Supplementary material for: Treatment outcomes of pre-surgical infant orthopedics in patients with non-syndromic cleft lip and/or palate: A systematic review and meta-analysis of randomized controlled trials
Source: PLoS One. 2017 Jul 24;12(7):e0181768. doi: 10.1371/journal.pone.0181768 (PMC5524403; doi:10.1371/journal.pone.0181768)
Supplement: S2 Table — (DOCX) [file pone.0181768.s004.docx]

**S2 Table. General characteristics of the studies included in the systematic review – Publications from the DUTCHCLEFT.**

| **Study & Intervention characteristics** | **Included outcomes** | **Additional information** |
| --- | --- | --- |
| **DUTCHCELFT** Netherlands **[Common items]**  **Group 1**: Passive PSIO appliance without external retention [Zurich / Hotz plate].  **Group 2**: No intervention. | **See details below in individual papers** | **A priori sample calculation:**  SNA difference of 3 degrees between the two groups (23 patients per group)  **Information on compliance:**  Yes |
| **Bongaarts et al., 2004 [29]** | **5-year-old index** (categorizing arch relationships using reference models)**, Huddart score** (dental arch relationships at the transverse planes)**, overjet, overbite, sagittal occlusion assessment** (scored for deciduous canines and second deciduous molars according to Angle classification)  **Assessment:** Impressions taken at 4 and 6 years of age and fabrication of casts | **Reliability of measurements:**  Examined |
| **Bongaarts et al., 2006 [32]** | **Maxillary arch dimensions - arch width, arch depth, arch length, arch form, and vertical position of the lesser segment, variables regarding cleft width** (alveolar cleft width, midpalatal cleft width, posterior cleft width at the tuberosity level), linear arch dimensions (anterior arch width, tuberosity width, total arch depth, total arch length, alveolar cleft margins length) and angular measurements (angulation of the greater alveolar segment in the transverse plane of space, angulation of the smaller alveolar segment in the transverse plane of space, vertical slope of the greater alveolar segment, vertical slope of the smaller alveolar segment).  **Contact and collapse of the alveolar segments** in maxillary casts at various ages**.**  **Assessment:** Impressions taken at 4, 6 years of age and plaster castes fabricated. (Maxillary casts analyzed using Reflex Microscope, Contact was scored as absent (0) or present (1), collapse scored as absent (0), slight (1), moderate (2), severe (3)) | **Reliability of measurements:**  Examined. The measurements in the vertical direction had low reliability. |
| **Bongaarts et al., 2008 [34]** | **Visual analog scores** of facial appearance from full face and cropped photographs (focusing on the mouth and nose area)  **Assessment:** 4 and 6 years of age. | **Reliability of measurements:**  Examined |
| **Bongaarts et al., 2009 [36]** | **Dentofacial cephalometric variables evaluation** (angular, linear and ratio variable representing soft and hard tissues, as well as, dental structures)  **Assessment:** 4 and 6 years of age.  (Lateral head films taken, landmarks digitized on scanned images using Viewbox, version 3.1.0.5, occlusion scored using 5-year old index, esthetics scored using visual analog scale on facial photographs) | **Reliability of measurements:**  Examined. The largest errors were found in measurements involving point A or ANS, or the soft tissues. The reliability was good to acceptable, except for two measurements: upper incisor to ANS-PNS angle and ANS-PNS – SN angle. These two measurements were excluded from further analysis. |

**S2 Table. General characteristics of the studies included in the systematic review – Publications from the DUTCHCLEFT. [Continued]**

| **Study & Intervention characteristics** | **Included outcomes** | **Additional information** |
| --- | --- | --- |
| **Konst et al., 1999 [22]** | **Analysis of prelexical utterances** by means of a perceptually based sensori-motoric classification system.  **Assessment:** 12 and 18 months.  (Sound production recorded for speech and language evaluation) | **Reliability of measurements:**  Examined |
| **Konst, 2000 [23]** | **Future need for speech therapy**  **Assessment:** 2.5 years.  (Five trained female speech therapists assessed children`s speech) | **Reliability of measurements:**  Examined |
| **Konst et al., 2003a [25]** | **Various speech characteristics:** Place of articulation, voice characteristics, nasalization, correctness of articulation, intelligibility, total impression  **Assessment:** 2.5 years.  (Five trained female speech therapists assessed children`s speech in a blinded perceptual rating procedure on a EAI scales (a seven-point scale) and a 10 point scale for total impression) | **Reliability of measurements:**  Examined |
| **Konst et al., 2003b [26]** | **Receptive language skills** (Mean length of utterances, mean length of longest utterances)  **Assessment:** 2, 2.5, 3 and 6 years.  (Reynell Developmental Language Scales Dutch Version test, and in 6-year-olds standardized Dutch language tests) | **Reliability of measurements:**  NR |
| **Konst et al., 2003c [27]** | **Phonological skills** (Number of acquired consonants, order of phonological development, use of phonological processes, and occurrence of nasal escape)  **Assessment: 2, 2.5, 3 years of age.**  (A system for assessing phonological development of Dutch children; Fonologische Analyse van het Nederlands: FAN) | **Reliability of measurements:**  Examined |
| **Konst et al., 2004 [30]** | **Cost-effectiveness of PSIO regarding speech intelligibility**  **Assessment: 2.5 years of age.**  (Intelligibility assessed by five trained speech therapists judging the total impression of speech quality on a 10-point equal-appearing interval scale and costs measured from a social view point in Euro) | **Reliability of measurements:**  NR |
| **Noverraz et al., 2015 [38]** | **Modified Huddart/Bodenham score**  **Assessment:** 9 and 12 years of age.  (Impressions taken at 9 and 12 years of age and maxillary casts fabricated) | **Reliability of measurements:**  Examined |
| **Prahl et al., 2001 [5]** | **Evaluation of maxillary arch dimensions** (Linear and angular maxillary dentoalveolar variables)  **Assessment:** 2, 15, 24, 48, 78 weeks  (Impressions taken at 2, 15, 24, 48, 78 weeks and plaster castes fabricated. 13 reference points were described and analyzed using Reflex Microscope technique and 15 dimensions were calculated as well as their increments between the ages) | **Reliability of measurements:**  Examined |

**S2 Table. General characteristics of the studies included in the systematic review – Publications from the DUTCHCLEFT. [Continued]**

| **Study & Intervention characteristics** | **Included outcomes** | **Additional information** |
| --- | --- | --- |
| **Prahl et al., 2003 [28]** | **Presence of contact and/or overlap (collapse) between cleft segments** in maxillary casts at various ages.  **Assessment:** 2, 15, 24, 48 and 78 weeks.  (Contact was scored as absent (0) or present (1), collapse scored as absent (0), slight (1), moderate (2), severe (3)) | **Reliability of measurements:**  Examined |
| **Prahl et al., 2005 [31]**  Feeding instructions given by the orthodontist (use of a squeeze bottle) | **Weight for age, length for age, weight for length, various feeding variables** (time per feeding (min), amount per feeding (mL) and feeding velocity (mL/min))  **Assessment:** 2, 3, 6, 15, 24 weeks  (Feeding log/questionnaire were given to the mothers at five different times, weight and length were measured according to national protocol by national infant consultation centers) | **Reliability of measurements:**  NR |
| **Prahl et al., 2006 [33]** | **Visual analog scores** and comparison scores to a reference photograph of facial appearance from full face and cropped photographs (focusing on the mouth and nose area)  **Assessment:** 18 months. | **Reliability of measurements:**  Examined |
| **Prahl et al., 2008 [35]** | **Various parameters investigating satisfaction in motherhood** (interaction and caretaking of the baby, coming and goings of the baby, motherhood and life outside and support).  **Assessment: 6, 24, 58 weeks.**  (Questionnaires, a 4-point scale was used: 1 = very satisfactory to 4 = very unsatisfactory) | **Reliability of measurements:**  Examined but the psychometric properties of the instrument not appropriately investigated. |
| **Severens et al., 1998 [21]** | **Short-term cost-effectiveness of PSIO** (time taken for the surgical lip closure procedure compared to medical and non medical costs until surgical lip closure)  **Assessment: 18 weeks.** | ***A priori* sample calculation:**  NR |

NR: Not Reported, EAI: equal-appearing interval
