# Supplementary material for: Treatment outcomes of pre-surgical infant orthopedics in patients with non-syndromic cleft lip and/or palate: A systematic review and meta-analysis of randomized controlled trials
Source: PLoS One. 2017 Jul 24;12(7):e0181768. doi: 10.1371/journal.pone.0181768 (PMC5524403; doi:10.1371/journal.pone.0181768)
Supplement: S3 Table — (DOCX) [file pone.0181768.s005.docx]

**S3 Table. General characteristics of the studies included in the systematic review – Remaining studies.**

| **Study & Intervention characteristics** | **Included outcomes** | **Additional information** |
| --- | --- | --- |

| **Chang et al., 2014 [37]**  Taiwan  **Group 1:**  Modified Figueroa NAM technique.  **Group 2:**  Modified Grayson NAM technique. | **Number of clinical visits, total costs, complications, and nasal symmetry** (Nostril height, nostril width, nostril sill height, nostril area)  **Assessment:**  Initial visit, after NAM but before surgery, 1 week after surgery, 6 months after surgery.  (Using standardized craniofacial photographs according to European Association of Cranio-Maxillo-Facial Surgery) | **A priori sample calculation:**  Nostril height was considered 1 year after surgery to be the primary outcome and 1-mm difference to be clinically significant.  **Reliability measurements:**  Examined |
| --- | --- | --- |
| **Masarei et al., 2007 [8]**  UK  **Group 1:**  Active or passive plate  **Group 2:**  No PSIO | **Oral motor skills during feeding** (at 3 months: Neonatal Oral Motor Assessment Scale; at 6 months: Schedule of Oral Motor Assessment)**, physiological measures of bottle feeding/sucking** (at 3 months Great Ormond Street Measurement of Infant Feeding - length of sucking bursts, peak-to-peak intervals, rate of sucking, suck-swallow rations and percentage pressure generation above baseline pressure in the feeding bottle)**, assessment of pharyngeal stage of swallowing** (at 3 months by videofluoroscopy)**, anthropometry** (at 3 and 6 months: weight, length, head circumference and Body Mass Index)  **Assessment:** 3 months, 12 months of age. | ***A priori* sample calculation:**  Change of 0.8 SD in the anthropometry z scores  **Reliability of measurements:**  NR |

UK: United Kingdom, NR: Not Reported, EAI: equal-appearing interval, NAM: Nasoalveolar molding.
