# Supplementary material for: Treatment outcomes of pre-surgical infant orthopedics in patients with non-syndromic cleft lip and/or palate: A systematic review and meta-analysis of randomized controlled trials
Source: PLoS One. 2017 Jul 24;12(7):e0181768. doi: 10.1371/journal.pone.0181768 (PMC5524403; doi:10.1371/journal.pone.0181768)
Supplement: S4 Table — (DOCX) [file pone.0181768.s006.docx]

**S4 Table. Participant characteristics of the studies included in the systematic review – Publications from the DUTCHCLEFT.**

| **Study** | **Inclusion & exclusion criteria** | **Number of patients randomized and analyzed** |
| --- | --- | --- |
| **DUTCHCELFT**  **[General assessment]** | **Inclusion criteria:** complete UCLP, infants born at term, both parents Caucasian and fluent in Dutch language, and trial entrance within 2 weeks after birth.  **Exclusion criteria:** other congenital malformations (except for syndactyly) and soft tissue bends. | **Randomized:** 54 children (13 M, 41 F)  **Group 1**: 27 (20 M 7 F)  **Group 2**: 27 (21 M 6 F) |
| **Bongaarts et al., 2004 [29]** | **[See first row]** | **Analyzed at age 4**:  **Group 1**: 22 analyzed  **Group 2**: 22 analyzed  **Analyzed at age 6**:  **Group 1**: 22 analyzed  **Group 2**: 23 analyzed |
| **Bongaarts et al., 2006 [32]** | **[See first row]** | **Analyzed at age 4**:  **Group 1**: 23 analyzed  **Group 2**: 22 analyzed  **Analyzed at age 6**:  **Group 1**: 22 analyzed  **Group 2**: 23 analyzed |
| **Bongaarts et al., 2008 [34]** | **[See first row]** | **Analyzed at age 4**:  **Group 1**: 24 analyzed  **Group 2**: 21 analyzed  **Analyzed at age 6**:  **Group 1**: 22 analyzed  **Group 2**: 24 analyzed |
| **Bongaarts et al., 2009 [36]** | **[See first row]** | **Analyzed at age 4**:  **Group 1**: 21 analyzed  **Group 2**: 20 analyzed  **Analyzed at age 6**:  **Group 1**: 21 analyzed  **Group 2**: 22 analyzed |
| **Konst et al., 1999 [22]** | **[See first row]** | **Analyzed at age 12 months**:  **Group 1**: 18 analyzed  **Group 2**: 18 analyzed  **Analyzed at age 18 months**:  **Group 1**: 19 analyzed  **Group 2**: 19 analyzed |

**S4 Table. Participant characteristics of the studies included in the systematic review – Publications from the DUTCHCLEFT. [Continued]**

| **Study** | **Inclusion & exclusion criteria** | **Number of patients randomized and analyzed** |
| --- | --- | --- |
| **Konst et al., 2000 [23]** | **[See first row]** | **Group 1**:  10 analyzed (8 M, 2 F)  **Group 2**:  10 analyzed (9 M, 1 F) |
| **Konst et al., 2003a [25]** | **[See first row]** | **Group 1**:  10 analyzed (8 M, 2 F)  **Group 2**:  10 analyzed (9 M, 1 F) |
| **Konst et al., 2003b [26]** | **[See first row]** | **Analyzed at age 2, 2.5, 3**:  **Group 1**: 6 analyzed  **Group 2**: 6 analyzed  **Analyzed at age 6**:  **Group 1**: 6 analyzed  **Group 2**: 5 analyzed |
| **Konst et al., 2003c [27]** | **[See first row]** | **Analyzed at age 2**:  **Group 1**: 9 analyzed  **Group 2**: 7 analyzed  **Analyzed at age 2.6**:  **Group 1**: 9 analyzed  **Group 2**: 9 analyzed |
| **Konst et al., 2004 [30]** | **[See first row]** | **Analyzed at age 2.5**:  **Group 1**: 10 analyzed (8 M, 2 F)  **Group 2**: 10 analyzed (9 M, 1 F) |
| **Noverraz et al., 2015 [38]** | **[See first row]** | **Analyzed at age 9**:  **Group 1**: 24 analyzed  **Group 2**: 21 analyzed  **Analyzed at age 12**:  **Group 1**: 22 analyzed  **Group 2**: 22 analyzed |
| **Prahl et al., 2001 [5]** | **[See first row]** | **Group 1**:  27 randomized (6 M, 21 F) (24 analyzed)  **Group 2**:  27 randomized (7 M, 20 F) (25 analyzed) |

**S4 Table. Participant characteristics of the studies included in the systematic review – Publications from the DUTCHCLEFT. [Continued]**

| **Study** | **Inclusion & exclusion criteria** | **Number of patients randomized and analyzed** | |
| --- | --- | --- | --- |
| **Prahl et al., 2003 [28]** | **[See first row]** | **At 2 weeks:**  **Group 1:** 24 analyzed  **Group 2:** 24 analyzed  **At 24 weeks:**  **Group 1:** 23 analyzed  **Group 2:** 24 analyzed  **At 58 weeks:**  **Group 1:** 16 analyzed  **Group 2:** 22 analyzed | **At 15 weeks:**  **Group 1:** 22 analyzed  **Group 2:** 25 analyzed  **At 48 weeks:**  **Group 1:** 22 analyzed  **Group 2:** 24 analyzed  **At 78 weeks:**  **Group 1:** 19 analyzed  **Group 2:** 20 analyzed |
| **Prahl et al., 2005 [31]** | **[See first row]** | **At 2 weeks:**  **Group 1:** 21 analyzed  **Group 2:** 22 analyzed  **At 6 weeks:**  **Group 1:** 20 analyzed  **Group 2:** 24 analyzed  **At 24 weeks:**  **Group 1:** 20 analyzed  **Group 2:** 21 analyzed | **At 3 weeks:**  **Group 1:** 16 analyzed  **Group 2:** 21 analyzed  **At 15 weeks:**  **Group 1:** 17 analyzed  **Group 2:** 24 analyzed |
| **Prahl et al., 2006 [33]** | **[See first row]** | **Group 1**:  20 analyzed (17 M, 3 F)  **Group 2**:  21 analyzed (17 M, 4 F) | |
| **Prahl et al., 2008 [35]** | **Inclusion criteria:** infants with UCLP or with ICP where the soft palate and at least two thirds of the hard palate was involved.  **Exclusion criteria:** infants who required cardiac surgery, neurological impairment, and syndrome known to adversely affect feeding and/ or growth. | **Analyzed at 6 weeks**:  **Group 1**: responses from 23 caregivers analyzed  **Group 2**: responses from 26 caregivers analyzed  **Analyzed at 24 weeks**:  **Group 1**: responses from 23 caregivers analyzed  **Group 2**: responses from 24 caregivers analyzed  **Analyzed at 58 weeks**:  **Group 1**: responses from 18 caregivers analyzed  **Group 2**: responses from 19 caregivers analyzed  [The total number of mothers who had been given the questionnaires were 49] | |

**S4 Table. Participant characteristics of the studies included in the systematic review – Publications from the DUTCHCLEFT. [Continued]**

| **Study** | **Inclusion & exclusion criteria** | **Number of patients randomized and analyzed** |
| --- | --- | --- |
| **Severens et al., 1998 [21]** | **[See first row]** | **Medical costs**  **Group 1**: 23 analyzed  **Group 2**: 20 analyzed  **Non-medical costs - Travel costs**  **Group 1**: 23 analyzed  **Group 2**: 20 analyzed  **Non-medical costs - Indirect costs**  **Group 1**: 15 analyzed  **Group 2**: 14 analyzed |

M: males, F: females, UCLP: unilateral cleft lip and palate.
