# Supplementary material for: Treatment outcomes of pre-surgical infant orthopedics in patients with non-syndromic cleft lip and/or palate: A systematic review and meta-analysis of randomized controlled trials
Source: PLoS One. 2017 Jul 24;12(7):e0181768. doi: 10.1371/journal.pone.0181768 (PMC5524403; doi:10.1371/journal.pone.0181768)
Supplement: S5 Table — (DOCX) [file pone.0181768.s007.docx]

**S5 Table. Participant characteristics of the studies included in the systematic review – Remaining studies.**

| **Study** | **Inclusion & exclusion criteria** | **Number of patients randomized and analyzed** | |
| --- | --- | --- | --- |
| **Chang et al., 2014 [37]** | **Inclusion criteria:** Infants with CUCLP, institutional review board-approved parent- or guardian-signed informed assent.  **Exclusion criteria:** Presence of other craniofacial anomalies, incomplete unilateral cleft lip and palate. | **Group 1:**  15 randomized (15 analyzed, 11 M 4 F)  **Group 2:**  15 randomized (15 analyzed, 8 M 7 F) | |
| **Massarei *et al.,* 2007 [8]** | **Inclusion criteria:** infants with UCLP or with ICP where the soft palate and at least two thirds of the hard palate was involved.  **Exclusion criteria:** infants who required cardiac surgery, neurological impairment, and syndrome known to adversely affect feeding and/ or growth. | **UCLP:** 33 subjects randomized  **Analyzed at 3 months of age:** | **ICP:** 16 subjects randomized |
|  |  | **Group 1**: 16 analyzed  **Group 2**: 16 analyzed | **Group 1:** 8 analyzed  **Group 2:** 8 analyzed |
|  |  | **Analyzed at 12 months of age:** | |
|  |  | **Group 1:** 13 analyzed  **Group 2:** 7 analyzed | **Group 1:** 7 analyzed  **Group 2:** 4 analyzed |

M: males, F: females, UCLP: Complete unilateral cleft lip and palate.
