# Supplementary material for: Treatment outcomes of pre-surgical infant orthopedics in patients with non-syndromic cleft lip and/or palate: A systematic review and meta-analysis of randomized controlled trials
Source: PLoS One. 2017 Jul 24;12(7):e0181768. doi: 10.1371/journal.pone.0181768 (PMC5524403; doi:10.1371/journal.pone.0181768)
Supplement: S6 Table — (DOCX) [file pone.0181768.s008.docx]

**S6 Table. Details of risk of bias assessment – Publications from the DUTCHCLEFT.** [Domains examined: 1: Random sequence generation 2: Allocation concealment, 3: Blinding of participants and personnel, 4: Blinding of outcome assessment, 5: Incomplete outcome data, 6: Selective outcome reporting, 7: Other potential threats to validity]

| **Study** | **Rating** | | **Reasons for rating** |
| --- | --- | --- | --- |
| **DUTCHCELFT**  **[General assessment]** | **1.** | Low | The exact method of randomization is mentioned. [“...subjects were randomly allocated to groups using the minimization methods...”] |
|  | **2.** | Low | Minimization is a convincing method of allocation concealment. [“...subjects were randomly allocated to groups using the minimization methods...”] |
|  | **3.** | Low | Blinding of the participants, caregivers and personnel was not possible. However, the review authors believe that the outcome is not likely to be influenced by lack of blinding. |
| **Bongaarts et al., 2004 [29]** | **4.** | Low | Statement that the investigator was blinded. No other reason to infer that the investigator could assume group allocation. [“To eliminate bias, all models were duplicated and trimmed in the same way. In this way the examiners were not able to identify a patient o r a cleft palate center.”] |
|  | **5.** | Unclear | Dropouts are described and explained. However, it is unclear how they could influence the various outcomes of the study. |
|  | **6.** | Low | All important outcomes are adequately reported. |
|  | **7.** | Unclear | It is unclear whether compliance or the other performed interventions until the age of 6 years old introduced some kind of bias. |
| **Bongaarts et al., 2006 [32]** | **4.** | Low | No reason to infer that the investigator could assume group allocation. [“To eliminate bias, all models were duplicated and trimmed in the same way. In this way the examiners were not able to identify a patient or a cleft palate center.”] |
|  | **5.** | Unclear | Dropouts are described and explained. However, it is unclear how they could influence the various outcomes of the study. |
|  | **6.** | Low | All important outcomes are adequately reported. |
|  | **7.** | Unclear | It is unclear whether compliance or the other performed interventions until the age of 6 years old introduced some kind of bias. |
| **Bongaarts et al., 2008 [34]** | **4.** | Low | No reason to infer that the investigators could assume group allocation. |
|  | **5.** | Unclear | Dropouts are described and explained. However, it is unclear how they could influence the various outcomes of the study. |
|  | **6.** | Low | All important outcomes are adequately reported. |
|  | **7.** | Unclear | It is unclear whether compliance or the other performed interventions until the age of 6 years old introduced some kind of bias. |

**S6 Table. Details of risk of bias assessment – Publications from the DUTCHCLEFT.** [Domains examined: 1: Random sequence generation 2: Allocation concealment, 3: Blinding of participants and personnel, 4: Blinding of outcome assessment, 5: Incomplete outcome data, 6: Selective outcome reporting, 7: Other potential threats to validity] [Continued]

| **Study** | **Rating** | | **Reasons for rating** |
| --- | --- | --- | --- |
| **Bongaarts et al., 2009 [36]** | **4.** | Low | No reason to infer that the investigators could assume group allocation. |
|  | **5.** | Unclear | Dropouts are described and explained. However, it is unclear how they could influence the various outcomes of the study. |
|  | **6.** | Low | All important outcomes are adequately reported. |
|  | **7.** | Unclear | It is unclear whether compliance, other performed interventions until the age of 6 years old, or uncertainly in landmark localization introduced some kind of bias. |
| **Konst et al., 1999 [22]** | **4.** | Low | No reason to infer that the investigators could assume group allocation. |
|  | **5.** | High | Dropouts are not fully described and explained. Moreover, it is unclear how they could influence the various outcomes of the study. |
|  | **6.** | Low | All important outcomes are adequately reported. |
|  | **7.** | Unclear | It is unclear how compliance may have influenced the results of the study. |
| **Konst et al., 2000 [23]** | **4.** | Low | No reason to infer that the investigators could assume group allocation. |
|  | **5.** | High | Dropouts are not fully described and explained. Moreover, it is unclear how they could influence the various outcomes of the study. |
|  | **6.** | Low | All important outcomes are adequately reported. |
|  | **7.** | Unclear | It is unclear how compliance may have influenced the results of the study. |
| **Konst et al., 2002 [24]**  **[only for future speech therapy need]** | **4.** | Low | No reason to infer that the investigators could assume group allocation. |
|  | **5.** | High | Dropouts are not fully described and explained. Moreover, it is unclear how they could influence the various outcomes of the study. |
|  | **6.** | Low | All important outcomes are adequately reported. |
|  | **7.** | Unclear | It is unclear how compliance may have influenced the results of the study. |

**S6 Table. Details of risk of bias assessment – Publications from the DUTCHCLEFT**. [Domains examined: 1: Random sequence generation 2: Allocation concealment, 3: Blinding of participants and personnel, 4: Blinding of outcome assessment, 5: Incomplete outcome data, 6: Selective outcome reporting, 7: Other potential threats to validity] [Continued]

| **Study** | **Rating** | | **Reasons for rating** |
| --- | --- | --- | --- |
| **Konst et al., 2003a [25]** | **4.** | Low | No reason to infer that the investigators could assume group allocation. |
|  | **5.** | High | Dropouts are not fully described and explained. Moreover, it is unclear how they could influence the various outcomes of the study. |
|  | **6.** | Unclear | An outcome included in the original thesis publication has been omitted from the article. |
|  | **7.** | Unclear | It is unclear how compliance may have influenced the results of the study. |
| **Konst et al., 2003b [26]** | **4.** | Low | No reason to infer that the investigators could assume group allocation. |
|  | **5.** | High | Dropouts are not fully described and explained. Moreover, it is unclear how they could influence the various outcomes of the study. |
|  | **6.** | Low | All important outcomes are adequately reported. |
|  | **7.** | Unclear | It is unclear whether compliance or the other performed interventions until the age of 6 years old introduced some kind of bias. |
| **Konst et al., 2003c [27]** | **4.** | Low | No reason to infer that the investigators could assume group allocation. |
|  | **5.** | High | Dropouts are not fully described and explained. Moreover, it is unclear how they could influence the various outcomes of the study. |
|  | **6.** | Low | All important outcomes are adequately reported. |
|  | **7.** | Unclear | It is unclear how compliance may have influenced the results of the study. |
| **Konst et al., 2004 [30]** | **4.** | Low | No reason to infer that the investigators could assume group allocation. |
|  | **5.** | High | Dropouts are not fully described and explained. Moreover, it is unclear how they could influence the various outcomes of the study. |
|  | **6.** | Low | All important outcomes are adequately reported. |
|  | **7.** | Unclear | It is unclear how compliance may have influenced the results of the study. |

**S6 Table. Details of risk of bias assessment – Publications from the DUTCHCLEFT.** [Domains examined: 1: Random sequence generation 2: Allocation concealment, 3: Blinding of participants and personnel, 4: Blinding of outcome assessment, 5: Incomplete outcome data, 6: Selective outcome reporting, 7: Other potential threats to validity] [Continued]

| **Study** | **Rating** | | **Reasons for rating** |
| --- | --- | --- | --- |
| **Noverraz et al., 2015 [38]** | **4.** | Low | Statement that the investigator was blinded. No other reason to infer that the investigator could assume group allocation. [“To eliminate bias, all models were duplicated and trimmed in the same way. In this way the examiners were not able to identify a patient or a cleft palate center.”] |
|  | **5.** | Unclear | Dropouts are described and explained. However, it is unclear how they could influence the various outcomes of the study. |
|  | **6.** | Unclear | Outcomes such as overjet, overbite and sagittal occlusion appearing in previous similar study from the same sample are missing. |
|  | **7.** | Unclear | It is unclear whether compliance or the other performed interventions until the age of 12 years old introduced some kind of bias. |
| **Prahl et al., 2001 [5]** | **4.** | Low | No reason to infer that the investigator could assume group allocation. [“Data entry and analysis were blinded. The orthodontists who treated the patients did not assess the maxillary dimensions.”] |
|  | **5.** | Low | Dropouts are described and explained. |
|  | **6.** | Low | All important outcomes are adequately reported. |
|  | **7.** | Unclear | It is unclear how compliance may have influenced the results of the study. |
| **Prahl et al., 2003 [28]** | **4.** | Low | No reason to infer that the investigator could assume group allocation. |
|  | **5.** | Low | Dropouts are described and explained. |
|  | **6.** | Low | All important outcomes are adequately reported. |
|  | **7.** | Unclear | It is unclear how compliance may have influenced the results of the study. |
| **Prahl et al., 2005 [31]** | **4.** | Low | No reason to infer that the investigator could assume group allocation. |
|  | **5.** | Unclear | Dropouts are described and explained. However, it is unclear how they could influence the various outcomes of the study. |
|  | **6.** | Low | All important outcomes are adequately reported. |
|  | **7.** | Unclear | It is unclear how compliance may have influenced the results of the study. |

**S6 Table. Details of risk of bias assessment – Publications from the DUTCHCLEFT**. [Domains examined: 1: Random sequence generation 2: Allocation concealment, 3: Blinding of participants and personnel, 4: Blinding of outcome assessment, 5: Incomplete outcome data, 6: Selective outcome reporting, 7: Other potential threats to validity] [Continued]

| **Study** | **Rating** | | **Reasons for rating** |
| --- | --- | --- | --- |
| **Prahl et al., 2006 [33]** | **4.** | Low | No reason to infer that the investigator could assume group allocation. |
|  | **5.** | Low | Dropouts are described and explained. |
|  | **6.** | Low | All important outcomes are adequately reported. |
|  | **7.** | Unclear | It is unclear how compliance may have influenced the results of the study. |
| **Prahl et al., 2008 [35]** | **4.** | Low | No reason to infer that the investigator could assume group allocation. |
|  | **5.** | Unclear | Dropouts are described and explained. However, it is unclear how they could influence the various outcomes of the study. |
|  | **6.** | Low | All important outcomes are adequately reported. |
|  | **7.** | Unclear | The study questionnaire had not been previously validated. |
| **Severens et al., 1998 [21]** | **4.** | Low | No reason to infer that the investigators could assume group allocation. |
|  | **5.** | High | Dropouts are not fully described and explained. Moreover, it is unclear how they could influence the various outcomes of the study. |
|  | **6.** | Low | All important outcomes are adequately reported. |
|  | **7.** | Low | The study appears to be free of other sources of bias. |
