# Supplementary material for: Treatment outcomes of pre-surgical infant orthopedics in patients with non-syndromic cleft lip and/or palate: A systematic review and meta-analysis of randomized controlled trials
Source: PLoS One. 2017 Jul 24;12(7):e0181768. doi: 10.1371/journal.pone.0181768 (PMC5524403; doi:10.1371/journal.pone.0181768)
Supplement: S7 Table — (DOCX) [file pone.0181768.s009.docx]

**S7 Table. Details of risk of bias assessment – Remaining studies.** [Domains examined: 1: Random sequence generation 2: Allocation concealment, 3: Blinding of participants and personnel, 4: Blinding of outcome assessment, 5: Incomplete outcome data, 6: Selective outcome reporting, 7: Other potential threats to validity]

| **Study** | **Rating** | | **Reasons for rating** |
| --- | --- | --- | --- |
| **Chang et al., 2014 [37]** | **1.** | Low | The exact method of randomization is mentioned. [“They were block randomized at a 1:1 ratio to undergo either modified Figueroa or modified Grayson nasoalveolar molding by an independent third-party specialized trials nurse using secure randomization envelopes.” |
|  | **2.** | Low | No statement on the envelopes being sequentially administered. No other reason to infer that the investigator could influence group allocation. [“by an independent third-party specialized trials nurse using secure randomization envelopes.”] |
|  | **3.** | Low | Blinding of the participants, caregivers and personnel was not possible. However, the review authors believe that the outcome is not likely to be influenced by lack of blinding. |
|  | **4.** | Low | Statement that the investigator was blinded. No other reason to infer that the investigator could assume group allocation. [“All assessors were blinded regarding the nasoalveolar molding technique that patients had been randomized to undergo; no breaks in the blind were reported.”] |
|  | **5.** | Low | No dropouts occurred. |
|  | **6.** | Low | All important outcomes are adequately reported. |
|  | **7.** | Low | The study appears to be free of other potential threats to validity. |
| **Masarei et al. 2007 [8]** | **1.** | Low | The exact method of randomization is mentioned. [“Minimization was therefore used to ensure that the Iwo groups contained similar numbers of first-born, later born, and male and female infants. Data for patient allocation were entered by the researcher using MINIM.”] |
|  | **2.** | Low | Minimization is a convincing method of allocation concealment. [“Minimization was therefore used to ensure that the Iwo groups contained similar numbers of first-born, later born, and male and female infants. Data for patient allocation were entered by the researcher using MINIM.”] |
|  | **3.** | Low | Blinding of the participants, caregivers and personnel was not possible. However, the review authors believe that the outcome is not likely to be influenced by lack of blinding. |
|  | **4.** | Low | No statement, but no reason to infer that the investigator could assume group allocation. |
|  | **5.** | Unclear | Dropouts are described and explained. However, it is unclear how they could influence the various outcomes of the study. |
|  | **6.** | Low | All important outcomes are adequately reported. |
|  | **7.** | Unclear | It is unclear how compliance may have influenced the results of the study. |
