# Supplementary material for: Treatment outcomes of pre-surgical infant orthopedics in patients with non-syndromic cleft lip and/or palate: A systematic review and meta-analysis of randomized controlled trials
Source: PLoS One. 2017 Jul 24;12(7):e0181768. doi: 10.1371/journal.pone.0181768 (PMC5524403; doi:10.1371/journal.pone.0181768)
Supplement: S8 Table — (DOCX) [file pone.0181768.s010.docx]

**S8 Table. Quality of available evidence for the outcomes of weight and height.**

| **Quality assessment** | | | | | | **№ of patients** | | **Effect** | **Quality** |
| --- | --- | --- | --- | --- | --- | --- | --- | --- | --- |
| **Studies** | **Risk of bias** | **Inconsistency** | **Indirectness** | **Imprecision** | **Other** | **PSIO** | **Control** | **Absolute (95% CI)** |  |
| **Weight** [follow up: around 12 months; assessed with: z scores] | | | | | | | | | |
| 2 | Serious^1^ | Not serious | Serious^2^ | Serious^3^ | None | 27 | 25 | MD **0.132 z scores higher** (0.364 lower to 0.628 higher) *p*=0.628 | ⨁◯◯◯  **VERY LOW** |
| **Height** [follow up: around 12 months; assessed with: z scores] | | | | | | | | | |
| 2 | Serious^1^ | Not serious | Serious^2^ | Serious^3^ | None | 27 | 25 | MD **0.007 z scores lower** (0.502 lower to 0.489 higher)  *p*=0.979 | ⨁◯◯◯  **VERY LOW** |

CI: Confidence interval; MD: Mean difference

^1^ Papers included were considered to be at unclear risk of bias. ^2^ Results were based on specific populations and treatment protocols. ^3^ The number of patients analyzed was limited.
