# Supplementary material for: Treatment outcomes of pre-surgical infant orthopedics in patients with non-syndromic cleft lip and/or palate: A systematic review and meta-analysis of randomized controlled trials
Source: PLoS One. 2017 Jul 24;12(7):e0181768. doi: 10.1371/journal.pone.0181768 (PMC5524403; doi:10.1371/journal.pone.0181768)
Supplement: S10 Table — (DOCX) [file pone.0181768.s012.docx]

**S10 Table. Quality of available evidence for selected cephalometric measurements.**

| **Quality assessment** | | | | | | **№ of patients** | | **Effect** | **Quality** |
| --- | --- | --- | --- | --- | --- | --- | --- | --- | --- |
| **Studies** | **Risk of bias** | **Inconsistency** | **Indirectness** | **Imprecision** | **Other** | **PSIO** | **Control** | **Absolute (95% CI)** |  |
| **SNA** [follow up: 6 years of age; assessed with: degrees (^o^)] | | | | | | | | | |
| 1 | Not serious | Not serious | Serious^1^ | Serious^2^ | None | 21 | 20 | MD **1.290 degrees (^o^) lower** (-3.858 lower to 1.278 higher) *p*=0.306 | ⨁⨁◯◯ **LOW** |
| **ANB** [follow up: 6 years of age; assessed with: degrees (^o^)] | | | | | | | | | |
| 1 | Not serious | Not serious | Serious^1^ | Serious^2^ | None | 15 | 16 | MD **1.000 degrees (^o^) lower** (-3.201 lower to 1.201 higher)  *p*=0.362 | ⨁⨁◯◯ **LOW** |

CI: Confidence interval; MD: Mean difference

^1^ Results were based on specific populations and treatment protocols. ^2.^ The number of patients analyzed was limited.
