# Supplementary material for: Treatment outcomes of pre-surgical infant orthopedics in patients with non-syndromic cleft lip and/or palate: A systematic review and meta-analysis of randomized controlled trials
Source: PLoS One. 2017 Jul 24;12(7):e0181768. doi: 10.1371/journal.pone.0181768 (PMC5524403; doi:10.1371/journal.pone.0181768)
Supplement: S11 Table — (DOCX) [file pone.0181768.s013.docx]

**S11 Table. Quality of available evidence for selected maxillary arch dentoalveolar variables.**

| **Quality assessment** | | | | | | **№ of patients** | | **Effect** | **Quality** |
| --- | --- | --- | --- | --- | --- | --- | --- | --- | --- |
| **Studies** | **Risk of bias** | **Inconsistency** | **Indirectness** | **Imprecision** | **Other** | **PSIO** | **Control** | **Absolute (95% CI)** |  |
| **Arch width (P2-P2’)** [follow up: 6 years of age; assessed with: mm] | | | | | | | | | |
| 1 | Not serious | Not serious | Serious^1^ | Serious^2^ | None | 22 | 22 | MD **0.980 mm lower** (-2.641 lower to 0.681 higher) *p*=0.240 | ⨁⨁◯◯ **LOW** |
| **Arch depth (I-TT’)** [follow up: 6 years of age; assessed with: mm] | | | | | | | | | |
| 1 | Not serious | Not serious | Serious^1^ | Serious^2^ | None | 15 | 16 | MD **0.110 mm higher** (-1.855 lower to 2.075 higher)  *p*=0.910 | ⨁⨁◯◯ **LOW** |

CI: Confidence interval; MD: Mean difference

^1^ Results were based on specific populations and treatment protocols. ^2.^ The number of patients analyzed was limited.
