# Supplementary material for: Treatment outcomes of pre-surgical infant orthopedics in patients with non-syndromic cleft lip and/or palate: A systematic review and meta-analysis of randomized controlled trials
Source: PLoS One. 2017 Jul 24;12(7):e0181768. doi: 10.1371/journal.pone.0181768 (PMC5524403; doi:10.1371/journal.pone.0181768)
Supplement: S15 Table — (DOCX) [file pone.0181768.s017.docx]

**S15 Table. Quality of available evidence for total cost of treatment by the orthodontist.**

| **Quality assessment** | | | | | | **№ of patients** | | **Effect** | **Quality** |
| --- | --- | --- | --- | --- | --- | --- | --- | --- | --- |
| **Studies** | **Risk of bias** | **Inconsistency** | **Indirectness** | **Imprecision** | **Other** | **PSIO** | **Control** | **Absolute** |  |
| **Speech intelligibility score** [follow up: 2.5 years of age; assessed with: points] | | | | | | | | | |
| 1 | Serious^1^ | Not serious | Serious^2^ | Serious^3^ | None | 10 | 10 | MD **1041 € higher** | ⨁◯◯◯  **VERY LOW** |

MD: Mean difference

^1^ The study was considered of high risk of bias. ^2^Results were based on specific populations and treatment protocols. ^3.^ The number of patients analyzed was limited.
