# Supplementary material for: Treatment outcomes of pre-surgical infant orthopedics in patients with non-syndromic cleft lip and/or palate: A systematic review and meta-analysis of randomized controlled trials
Source: PLoS One. 2017 Jul 24;12(7):e0181768. doi: 10.1371/journal.pone.0181768 (PMC5524403; doi:10.1371/journal.pone.0181768)
Supplement: S16 Table — (DOCX) [file pone.0181768.s018.docx]

**S16 Table. Quality of available evidence for nostril height and nostril width ratios.**

| **Quality assessment** | | | | | | **№ of patients** | | **Effect** | **Quality** |
| --- | --- | --- | --- | --- | --- | --- | --- | --- | --- |
| **Studies** | **Risk of bias** | **Inconsistency** | **Indirectness** | **Imprecision** | **Other** | **mF** | **mG** | **Absolute (95% CI)** |  |
| **Nostril Height ratio** [follow up: approximately 9 months of age; assessed with: points] | | | | | | | | | |
| 1 | Not serious | Not serious | Serious^1^ | Serious^2^ | None | 15 | 15 | MD **0.000 points** (-0.097 lower to 0.097 higher) *p*=0.934 | ⨁⨁◯◯ **LOW** |
| **Nostril width ratio** [follow up: approximately 9 months of age; assessed with: points] | | | | | | | | | |
| 1 | Not serious | Not serious | Serious^1^ | Serious^2^ | None | 15 | 15 | MD **0.286 points higher** (-0.203 lower to 0.663 higher)  *p*=0.313 | ⨁⨁◯◯ **LOW** |

mF: modified Figueroa technique; mG: modified Grayson technique; CI: Confidence interval; MD: Mean difference

^1^ Results were based on specific populations and treatment protocols. ^2.^ The number of patients analyzed was limited.
