# Supplementary material for: Treatment outcomes of pre-surgical infant orthopedics in patients with non-syndromic cleft lip and/or palate: A systematic review and meta-analysis of randomized controlled trials
Source: PLoS One. 2017 Jul 24;12(7):e0181768. doi: 10.1371/journal.pone.0181768 (PMC5524403; doi:10.1371/journal.pone.0181768)
Supplement: S17 Table — (DOCX) [file pone.0181768.s019.docx]

**S17 Table. Quality of available evidence for cost comparison between different nasoalveolar molding techniques.**

| **Quality assessment** | | | | | | **№ of patients** | | **Effect** | **Quality** |
| --- | --- | --- | --- | --- | --- | --- | --- | --- | --- |
| **Studies** | **Risk of bias** | **Inconsistency** | **Indirectness** | **Imprecision** | **Other** | **mF** | **mG** | **Absolute (95% CI)** |  |
| **Total costs of treatment for parents/caregiver** [follow up: approximately 9 months of age; assessed with: points] | | | | | | | | | |
| 1 | Not serious | Not serious | Serious^1^ | Serious^2^ | None | 15 | 15 | MD **81.00 US$ higher** (-115.93 lower to 277.93 higher) *p*=0.357 | ⨁⨁◯◯ **LOW** |
| **Total cost of treatment for national insurance** [follow up: approximately 9 months of age; assessed with: points] | | | | | | | | | |
| 1 | Not serious | Not serious | Serious^1^ | Serious^2^ | None | 15 | 15 | MD **600.00 US$ higher** (-713.1 lower to 1913.1 higher)  *p*=0.357 | ⨁⨁◯◯ **LOW** |

mF: modified Figueroa technique; mG: modified Grayson technique; CI: Confidence interval; MD: Mean difference

^1^ Results were based on specific populations and treatment protocols. ^2.^ The number of patients analyzed was limited.
